# A Comprehensive Survey on Online AutoML and Adversarial Robustness for IoT and EV Charging Network Security

**DOI:** 10.3390/s26123886

**Published:** 2026-06-18

**Authors:** Wajiha Zaheer, Chukwunonso Henry Nwokoye, Seyedeh Negar Afrasiabi, Khalil El-Khatib, Li Yang

**Affiliations:** Faculty of Business and Information Technology, Ontario Tech University, Oshawa, ON L1G 0C5, Canada

**Keywords:** online stream learning, automated machine learning (AutoML), adversarial machine learning, Internet of Things (IoT), Electric Vehicles Charging Networks (EVCN)

## Abstract

The increasing deployment of IoT-enabled electric-vehicle charging networks has created a rapidly evolving cyber–physical environment in which security mechanisms must operate amid ever-changing data patterns and resource constraints. In these environments, static Machine Learning (ML) pipelines are often insufficient because they struggle to adapt to concept drift issues, emerging attacks, and real-time operational requirements. We analyzed cybersecurity vulnerabilities, challenges of conventional ML approaches, and the possibilities of AI-powered, adaptive security measures. This paper examines Online AutoML and its advantages, including automated adaptation to streaming data, reduced human intervention, and privacy-preserving, resource-aware learning. Furthermore, this paper discusses adversarial attacks and defences in Online AutoML systems, highlighting the need for frameworks that jointly address concept drift, scalability, privacy, and adversarial threats. Finally, this study emphasizes the importance of establishing comprehensive public benchmarks for Online AutoML research.

## 1. Introduction

Over the past decade, machine learning (ML) has been extensively integrated into numerous practical applications, including medical diagnosis, financial fraud detection, recommender systems, and industrial process control, among others [1,2,3]. Despite its revolutionary influence, the endeavour to construct high-performing ML models remains intricate and resource-intensive. The conventional ML workflow requires substantial human expertise and entails iterative manual interventions across multiple phases, including preprocessing, feature engineering, model selection, and hyperparameter tuning/optimization [4,5]. This reliance on specialized knowledge not only impedes rapid deployment but also constrains accessibility for individuals lacking expertise [5,6]. Furthermore, traditional models often assume a stationary data environment and struggle to adapt when deployed in live, dynamic streaming contexts, as exemplified by Internet of Things (IoT) systems, where data distributions may evolve. These obstacles underscore the growing need for an automated ML development pipeline [7,8]. Automating the design and optimization processes for ML models can significantly improve the scalability, reproducibility, and responsiveness of intelligent systems. This need has driven the emergence of AutoML, which seeks to reduce barriers to entry and expedite innovation by automating fundamental components of the ML workflow. Section 9 contains the list of acronyms and their full meanings.

AutoML democratizes machine learning by automating algorithm selection and hyperparameter tuning for models within the pipeline. With AutoML’s ability to lower the barrier to entry and accelerate model development, it is increasingly being integrated into real-world systems across sectors such as medicine, finance, cybersecurity, and the Industrial Internet of Things (IIoT) [5,6].

IoT systems are networks of interconnected endpoints (devices) that detect, communicate, and share data to enable intelligent services across sectors such as healthcare, smart cities, and industrial systems [9]. These environments are characterized by extensive, diverse, and resource-constrained devices that perpetually produce streaming data. Sicari et al. [9] emphasize that IoT systems face major privacy, security, and trust issues, largely due to their decentralized architecture and constrained computational resources.

Moreover, IoT data are dynamic [8], with shifting patterns arising from real-time environmental changes and user behaviours, rendering conventional static batch learning methods insufficient. This has prompted the implementation of online learning methods, thereby enabling ongoing adaptive model enhancements in response to streaming data and concept drift. However, security risks such as botnets, device hijacking, denial-of-service (DoS) and distributed denial-of-service (DDoS) attacks [10] directly affect online ML models deployed in IoT environments, as corrupted data can degrade model reliability and accuracy. As a result, there is increasing interest in combining online learning and AutoML to facilitate adaptive model selection, hyperparameter optimization, and robustness against emerging threats in IoT systems.

Among these dynamic IoT applications, Electric Vehicle (EV) charging networks are critical because they integrate cyber, physical, energy, and operational domains within a single connected environment. EVs have become a fundamental component of this shift, providing a means for low-carbon transportation and facilitating enhanced energy integration and grid adaptability. This transition is evident in the rapid increase in global EV adoption: sales of lighter-duty EVs surpassed 10 million in 2022, and sales in the initial quarter of 2023 exceeded those of the corresponding period in 2022 by 25% [11]. By the end of 2022, there were roughly 2.7 million public charging facilities globally, with over 900,000 constructed that year, representing approximately 55% growth over 2021 levels [12]. The growing incorporation of EV charging facilities into smart grid frameworks has led to the emergence of new communication protocols, particularly the Open Charge Point Protocol (OCPP) and ISO 15118 [13], which regulate interactions between EVs, charging infrastructure, and back-end control platforms, while facilitating demand response, remote surveillance, and plug-and-charge authentication [14]. The contemporary EV charging infrastructure is inherently IoT-enabled, comprising interconnected controllers, sensors, and communication user interfaces (UIs) that enable real-time data exchange between charging equipment and centralized management platforms [15]. This IoT connectivity enables sophisticated load control, billing, user authentication, and grid services, including Vehicle-to-Grid (V2G) energy transfer [15]. A burgeoning body of research has employed ML and DL methods for intrusion detection and anomaly identification in Electric Vehicle Charging Station (EVCS) contexts [16,17]. Notwithstanding these advancements, the convergence of online learning adaptability, AutoML, and adversarial robustness in EV charging networks remains a relatively unexplored domain, motivating the survey presented in this paper.

Adversarial Machine Learning (AML) is a field that examines the vulnerability of ML models to intentionally crafted inputs, known as adversarial examples, that aim to induce incorrect predictions or conclusions [18]. The domain has seen significant advances as ML-driven applications expand into safety-critical sectors, such as healthcare, autonomous vehicles, and smart grid infrastructure. A fundamental understanding of AML is that even highly effective ML systems are vulnerable to being systematically misled by small, meticulously designed perturbations to inputs, manipulations that can be undetectable to the human eye yet consistently result in misclassification [19]. In the realm of cybersecurity, tangible adversaries with specific aims exist. AML research is directly relevant to the implementation of malware classification and intrusion detection systems [20]. In online learning, where models are constantly updated using fresh data streams, the threat landscape considerably broadens: attackers might introduce contaminated samples into the incoming streams of data to progressively degrade or alter model performance as time passes [21]. The interplay between online adaptability and adversarial robustness is a significant barrier to securing ML-driven, learning-based systems in evolving, IoT-connected environments, such as EV charging networks. This survey examines that intersection in depth.

Recent advancements have focused on integrating online learning with AutoML to extend its utility in dynamic settings [22]. Online learning enables models to learn and update incrementally from live streaming data. This is particularly critical in IoT environments, where traditional offline learning methods are often ineffective, and data are non-stationary, and concept/data drifts are prevalent. As a result, online learning approaches are needed to learn from dynamic data streams and to continuously address concept drift. Online AutoML systems [22,23] not only reduce human and time-overhead costs but also maintain performance in environments where manual model iteration would be infeasible due to the scale, complexity, or speed of data generation.

While Online AutoML offers considerable advantages, it also introduces security concerns that warrant attention. The continuous processing of live data and the complexity of autonomous pipelines make Online AutoML systems inherently vulnerable, providing adversaries with numerous attack vectors against model selection, hyperparameter tuning, and pipeline configuration. Research indicates that Neural Architecture Search (NAS)-generated models, which are integral to many AutoML frameworks, are more vulnerable to various malicious manipulations, including adversarial evasion, model poisoning, and functionality stealing [24]. The risk is particularly acute in IoT settings, where adversaries can manipulate streaming data to subvert the learning process, degrade models, or trigger costly retraining cycles. Studies have highlighted the loopholes of deep learning-based malware threat-hunting models in IoT environments, particularly in industrial contexts, where adversarial attacks can significantly reduce model accuracy [25]. However, the adversarial robustness of Online AutoML systems remains underexplored, despite their growing relevance for safety-critical applications.

While prior surveys have made significant strides in exploring AutoML frameworks [5,26] and IoT-specific challenges, including concept drift and resource constraints [8,27], they typically address these difficulties in isolation. In contrast, our work explicitly focuses on the adversarial robustness of Online AutoML systems operating in dynamic, security-critical IoT environments, where the risks of poisoning, evasion, and concept-drift manipulation are heightened. To the best of our knowledge, no existing survey synthesizes how Online AutoML frameworks respond to adversarial threats while maintaining performance in real-time edge deployments. We aim to bridge this critical gap by providing a focused review of both vulnerabilities and emerging defences, thereby contributing to the development of trustworthy and resilient Online AutoML for mission-critical IoT systems.

This literature review focuses on understanding and answering the following questions:

RQ1: What cybersecurity and operational challenges in IoT and EV charging networks motivate the need for Online AutoML and adaptive ML-based security mechanisms?
RQ2: How do Online AutoML frameworks support adaptive security in dynamic IoT and EV charging environments, particularly under concept drift, resource constraints, and streaming data conditions?
RQ3: What adversarial threats and vulnerabilities affect Online AutoML systems, and how do its core components influence these risks?
RQ4: What defence strategies, evaluation practices, and future research directions are needed to develop robust, privacy-preserving, scalable, and practically deployable Online AutoML systems for IoT and EV charging networks?

Our motivation is rooted in the fact that EVCN has created a large-scale, dynamic cyber–physical environment characterized by continuous data streams, tight coupling with critical infrastructure, and increasing exposure to cyber threats. Despite progress in Online ML and AutoML for handling non-stationary data and reducing manual model engineering, existing studies remain fragmented, typically addressing adaptability, automation, or security in isolation. This work is motivated by the need to consolidate these perspectives and bridge the gap between adaptive learning and adversarial robustness in EV–IoT systems. It aims to support the development of adaptive, automated, and security-aware learning frameworks that ensure robust, resilient intelligence in EV charging infrastructure under dynamic and adversarial conditions.

This survey paper makes four main contributions. First, it describes the foundations of Online AutoML for streaming IoT and EV-charging environments. Second, it analyzes how adversarial attacks interact with key components of Online AutoML, including drift handling, model selection, and hyperparameter optimization. Third, it reviews defence strategies that can be adapted to Online AutoML pipelines. Fourth, it identifies open challenges in robustness, privacy, benchmarking, and deployment for safety-critical IoT and EV charging systems.

This paper is organized as follows: Section 2 and Section 3 presents the challenges and gaps in EVCN cybersecurity ecosystem. Section 4 introduces Online AutoML, including its foundations, current studies, and the role of online learning in handling heterogeneity and concept drift. Section 5 presents the architecture of Online AutoML frameworks. Section 6 reviews key AutoML libraries and frameworks relevant to IoT-based intrusion detection and streaming environments. Section 7 and Section 8 discuss adversarial vulnerabilities, research gaps, and future directions for secure and robust Online AutoML systems. Finally, Section 9 concludes the survey paper.

## 2. Cybersecurity Challenges in EVCN

The IoT has transformed connectivity across smart cities, industrial infrastructures, healthcare systems, and transportation networks [28,29]. However, this rapid growth of IoT has simultaneously produced a highly complex, multi-dimensional security landscape, with inherent architectural and systemic vulnerabilities. Device heterogeneity, constrained computational and energy resources, diverse operating environments, and geographically distributed deployments collectively hamper the adoption of standardized security protocols [28,29]. Consequently, IoT ecosystems exhibit heightened exposure to cyber–physical risks, in which compromises at individual devices or network nodes can propagate, potentially disrupting critical infrastructure and undermining system-wide reliability and resilience [30].

IoT systems face inherent structural challenges that distinguish them from traditional IT environments: devices often operate with severely constrained computational resources, rendering the implementation of conventional security measures impractical [10]. Many IoT deployments use heterogeneous hardware and software from multiple vendors, each with distinct security capabilities, update mechanisms, and operating systems, creating fragmented ecosystems in which standardized security protocols are difficult to enforce. The distributed and often remote nature of IoT deployments further complicates security monitoring, maintenance, and the timely application of firmware patches [31,32].

The attack surface in IoT environments spans multiple layers. First, at the communication layer, communication protocols often lack proper encryption or rely on outdated standards. Second, at the authentication layer, many devices rely on weak or default credentials that users rarely change after installation. Third, at the firmware and software layer, updates are frequently delivered insecurely and without proper validation mechanisms. Finally, at the physical layer, direct device access enables hardware-level attacks, such as rootkit implantation and component tampering [32,33,34].

A compromise at one layer may propagate across dependent systems, disrupting not only individual IoT devices but also broader networks and critical infrastructure [32]. The severity of this risk is underscored by Palo Alto Networks’ Unit 42 IoT Threat Report, which found that approximately 98% of IoT device traffic is transmitted without encryption, while 57% of IoT devices exhibit medium- or high-severity vulnerabilities [35]. The threat landscape is further complicated by AI-driven botnets that autonomously discover and exploit insecure IoT devices at scale [36]. These systemic IoT security limitations are particularly critical in EV charging infrastructure, where cyberattacks may directly affect both energy delivery and transportation operations.

### 2.1. EV Security Challenges

These generalized IoT security challenges are especially urgent in electric vehicle charging networks, where the convergence of energy infrastructure, transportation systems, and real-time IoT coordination creates significant systemic cybersecurity risks. The global expansion of Electric Vehicles (EVs) and IoT infrastructure represents one of the most rapid technological transformations of the past decade. EV charging networks are scaling at linear rates, especially in urban areas [37], supported by widespread IoT integration for real-time monitoring and predictive management [38,39]. Studies report substantial increases in both station counts and utilization rates, with European deployment expanding from 1.6 to 7.2 Battery Electric Vehicles (BEVs) per charging point between 2015 and 2020 [40]. Similarly, charging utilization rates and transaction frequencies have increased year over year, reflecting surging adoption and dependence on connected systems [41].

At large-scale deployment, EV charging demand may increase peak electricity demand by 35–51%. This exponential scaling, however, introduces serious systemic and cybersecurity challenges. If coordinated smart charging and IoT-enabled security control are not applied, this increase may be reduced to 30–41% [42]. These metrics show that security, reliability, and trust in IoT-enabled EV systems are not merely technical issues; they also directly affect energy efficiency, cost optimization, and grid stability. When IoT edge devices or back-end systems are compromised, overall system efficiency may deteriorate substantially, operational costs may increase, and safety risks may propagate across the network.

To address these concerns, the automotive and industrial sectors employ several cybersecurity and safety standards. ISO 26262 [43] defines functional safety requirements for electrical and electronic vehicle systems, while IEC 62443 [44] focuses on industrial control system security. Additional guidance is provided through SAE J3061 [45], ISO/SAE 21434 [46], and NIST SP 800-82 [47] for critical infrastructure protection [48,49]. These standards establish baseline requirements for secure communication, system resilience, firmware integrity, and risk management. Despite these frameworks and international standards, EV charging infrastructure continues to exhibit widespread vulnerabilities across communication protocols, cloud management systems, firmware update mechanisms, authentication frameworks, and physical hardware interfaces. Existing standards primarily provide generalized cybersecurity guidance and often lack domain-specific protections tailored to EV charging ecosystems, particularly for charger-to-grid communication, OCPP security, Over-The-Air (OTA) firmware update validation, and IoT-enabled charging coordination.

### 2.2. Common Cyberattacks in EV Charging Infrastructure

The most frequently reported attacks against EV charging infrastructure involve Man-in-the-Middle (MITM) exploitation of unencrypted or improperly authorized Open Charge Point Protocol (OCPP) sessions, denial-of-service and denial-of-charge attacks, which can escalate into false-data injection against charging telemetry, malicious firmware updates, credential spoofing, session hijacking, and remote exploitation of EV Charging Management Systems (EVCMSs) [50,51,52].

1. Communication Protocols (OCPP, PLC): Communication-layer attacks commonly target insecure OCPP and Power Line Communication (PLC) channels. Several studies demonstrate that sensitive information such as billing records, MAC addresses, authentication tokens, and telemetry data may be transmitted over insecure or weakly encrypted communication links, enabling interception, replay attacks, and session manipulation [53]. Vulnerabilities in the OCPP have been identified, revealing more than 12 zero-day flaws that enable MITM, Denial-of-Service (DoS), and data poisoning attacks. Such exploits not only disrupt charging operations but can also interfere with grid communication and compromise user privacy [52].
2. Back-end and Management Systems: Cloud-based management systems and mobile applications further increase the attack surface. Vulnerabilities such as SQL injection (SQLi), Cross-Site Scripting (XSS), Cross-Site Request Forgery (CSRF), weak authentication mechanisms, and insecure session management allow attackers to hijack charging sessions, manipulate charging behaviour, deploy malicious firmware, or gain unauthorized access to charging infrastructure management systems. These weaknesses allow attackers to hijack sessions, manipulate charging behaviour, or even install malicious firmware, exposing both personal data and grid control systems [50,52]. Firmware and hardware components also remain highly exposed. Insecure Over-the-Air (OTA) update mechanisms that lack cryptographic integrity verification may enable the deployment of persistent malware and remote code execution. Similarly, exposed debugging interfaces such as USB, UART, JTAG, and SWD ports may enable unauthorized firmware extraction, system reflashing, or hardware-level compromise [48].
3. Authentication and Authorization Failures: The most common cause of compromise is due to authentication and authorization failures [49]. Studies [32,50] show weak or absent user/vehicle verification mechanisms across multiple vendors. Inadequate access controls enable remote hijacking of charging sessions and manipulation of Vehicle-to-Grid (V2G) operations, thereby threatening grid stability and user safety. Additionally, electromagnetic emissions from Power Line Communication (PLC) hardware may reveal identifiers and billing information, enabling nearby attackers to infer sensitive operational data or to interfere with control signals [51].

These attacks form a highly interconnected cyber–physical attack surface capable of disrupting charging operations, compromising user privacy, destabilizing smart-grid coordination, and enabling large-scale attacks against connected transportation infrastructure.

### 2.3. Mapping EV Charging Attacks to MITRE ATT&CK, CWEs, and CVEs

Large-scale empirical studies demonstrate the operational relevance of attack mappings for risk and asset management within the industry to mitigate vulnerabilities with the EV ecosystem. Reference [50] reported widespread exposure of EV charging management systems and observed that approximately 24% of evaluated systems retained insecure default configurations resembling Mirai-style attack vectors. Similarly, reference [51] demonstrated practical exploitation of OCPP 1.6 implementations through MITM attacks, malicious firmware deployment, remote code execution, and denial-of-service attacks against operational Electric Vehicle Supply Equipment (EVSE) hardware. The taxonomy of recurring attack patterns identified in EV charging ecosystems has been built and organized around EVSE/EVCS components and protocols rather than a full ATT&CK matrix mapping [48,49]. However, these adversarial behaviours can be defined within the MITRE ATT&CK for ICS frameworks. These attacks span multiple ATT&CK tactics, including initial access, credential access, persistence, privilege escalation, command-and-control manipulation, and denial-of-service operations. Refernence [48] associate EV charging vulnerabilities with Common Weakness Enumerations (CWEs) that can be associated with publicly disclosed Common Vulnerabilities and Exposures (CVEs) and MITRE ATT&CK Attack IDs, enabling standardized characterization of attack severity, exploitability, and mitigation requirements.

Table 1 summarizes representative EV charging threats alongside their corresponding CWE classifications [48], representative CVEs, and associated MITRE ATT&CK techniques. For example, unencrypted OCPP communication sessions correspond to CWE-319 (Cleartext Transmission of Sensitive Information) and align with MITRE ATT&CK attack ID T1557 (Adversary-in-the-Middle). Similarly, insecure OTA firmware updates map to CWE-494 (Download of Code Without Integrity Check), which aligns with supply-chain compromise and ingress tool transfer techniques. These MITRE ATT&CK attack IDs can be used to map attack vectors and identify, mitigate, and defend against common attacks on EVCNs.

## 3. Frameworks and Standards for Evaluating EV Security Mechanisms

Beyond identifying vulnerabilities and attack vectors, several frameworks have been proposed to evaluate the resilience and effectiveness of EV charging security mechanisms. Cyber resilience governance increasingly relies on structured assessment models to systematically evaluate operational preparedness, threat mitigation capabilities, and infrastructure robustness.

Frameworks such as the Cyber Resilience Progression Model (CRPM) and the ICS Cyber Resilience Assessment Tool (ICSCRAT) provide methodologies for assessing resilience maturity within critical infrastructure environments while aligning with standards including NIST SP 800-82 [47], NIST 7628 [54], and ISO/IEC 27001 [49,55]. Similarly, MITRE TARA-based methodologies have been applied to identify critical EV charging assets, evaluate attack feasibility, and prioritize mitigation strategies [49]. However, existing evaluation frameworks primarily focus on static security controls, compliance assessment, and traditional risk management practices. Modern EV charging ecosystems generate highly dynamic and non-stationary data streams influenced by charging behaviour, IoT coordination, firmware updates, network traffic variability, and evolving attack strategies. Consequently, conventional rule-based defences and static IDSs may fail to adapt effectively to emerging threats and concept drift.

A single compromise, such as data manipulation or protocol injection, can cascade into widespread disruptions, ranging from charging stoppages to grid destabilization. These limitations motivate the need for more advanced end-to-end security mechanisms capable of real-time adaptation and autonomous threat detection. ML-based IDs therefore emerged as promising approaches for continuously monitoring EV charging environments, identifying anomalous behaviour, and adapting detection models dynamically with minimal human intervention.

### 3.1. AI-Driven Security Measures for IoT and EV Charging Networks

ML has become a central enabler of security in information technology (IT)/operation technology (OT)/IoT environments due to its ability to model complex patterns, intake high-volume data, and identify beyond predefined attack signatures. Unlike rule-based mechanisms, ML-driven security approaches can detect zero-day threats. Table 2 contains several attack vectors in IoT and EV charging networks as well as their relevance for ML-powered security.

Anomaly and Intrusion Detection Systems (IDSs) are among the most widely studied ML-based security mechanisms in cybersecurity. IDS models use supervised methods, such as support vector machines (SVMs), random forests (RFs), and deep neural networks, which are commonly used when labelled data are available. In contrast, unsupervised and semi-supervised techniques, including autoencoders, clustering methods, and isolation-based models, are favoured for detecting novel or zero-day attacks by learning normal behaviour profiles [56,57]. These approaches are typically deployed at gateways or edge nodes to monitor device and network activity in real time. Also, ML is widely used for detecting malware and firmware attacks in IoT devices. Static and dynamic analysis methods leverage ML models to identify malicious patterns in firmware binaries, system calls, or execution traces, enabling detection of attacks that evade traditional signature-based defences [58,59]. Deep learning models have proven particularly effective at capturing complex temporal and structural features in such data.

Another important application is behavioural profiling and device fingerprinting, where ML models learn device-specific communication and operational patterns to detect spoofing, impersonation, or device compromise. These methods are especially valuable in IoT settings where conventional authentication mechanisms are limited or infeasible [56]. Related work explores ML-enhanced authentication and access control, using contextual and behavioural features to provide adaptive or continuous verification and detect compromised credentials [58].

ML is further used for traffic classification, botnet detection, and coordinated attack identification, enabling early detection of large-scale threats such as DDoS attacks through flow-level and temporal analysis [57,59]. More recent studies investigate automated response and mitigation, where ML models dynamically select countermeasures, such as traffic throttling or device isolation, in response to detected threats [58]. Several studies in IoT and network security have shown that ML-based security measures substantially improve detection performance compared with traditional signature or rule-based methods. For instance, frameworks such as Mateen [60] and AOC-IDS [61] achieve detection accuracies of 97–98% for known threats, whereas traditional methods typically exhibit lower effectiveness against complex network traffic and evolving attacks. Moreover, ML approaches reduce false negatives for zero-day attacks, with adaptive and ensemble-based models achieving 89.6% detection of novel threats [62], a task that conventional signature-based systems often fail at. These results indicate that ML-based systems can generalize better to previously unseen attack patterns and provide enhanced coverage and responsiveness in dynamic IoT and network environments.

EVCNs are essential elements of contemporary smart grid systems, facilitating the extensive adoption of electric vehicles [11] while harmonizing energy consumption and availability [15]. Nonetheless, these systems are increasingly subjected to cyberattacks that exploit weaknesses in the OCPP [52,63], a widely used protocol for communication between EVCSs and centralized control platforms. OCPP vulnerabilities enable attackers to tamper with charging operations, modify billing information, or gain unauthorized access to the charging architecture [14]. These attacks pose significant threats to both the economic integrity of EVCN providers and the reliability of the grid, as coordinated hostile actions could induce abrupt load surges or interrupt energy distribution [15].

ML-inspired security methods have emerged as an effective approach for mitigating these threats by identifying unusual trends in charging behaviour [17]. By examining live and historical charging data, these models can detect anomalous behaviour suggestive of fraud, such as recurrent excessive charging by an individual user or inconsistencies between energy consumption and billing history [64]. Likewise, irregular load patterns indicative of grid exploitation can be identified by examining discrepancies between actual and anticipated energy consumption across various charging stations. Integrating ML-powered anomaly detection into electric vehicle charging networks improves operational safety and dependability, facilitates preventive attack response, and preserves confidence in the intelligent grid environment [65]. These methodologies are particularly advantageous in large-scale installations, where physical supervision is impractical, and the intricate interconnections among consumers, charging stations, and grid systems require intelligent, digital management [37].

Various solutions have been developed to mitigate attacks against OCPP in EVCN, leveraging ML-powered anomaly and IDSs. One method uses supervised ML algorithms [16] trained on labelled charging periods to distinguish between normal user behaviour and harmful activities, such as unlawful session activation or energy value manipulation. In addition, unsupervised models, such as clustering and autoencoder approaches, can identify previously unrecognized abnormalities, making them particularly useful for mitigating zero-day vulnerabilities in OCPP and innovative billing malpractice tactics [63]. By continuously monitoring the temporal and geographic dynamics of charging behaviour, these ML models provide real-time notifications of suspicious activity, enabling operators to respond before fraud or grid interruptions occur.

One defensive technique is to use hybrid systems that integrate ML-powered anomaly detection [16] with rule-driven access restrictions and communication protocol-layer upgrades to enhance security. Anomaly detection [60,66,67] can spot anomalous behaviour at specific EVCSs, which is subsequently cross-verified with grid-level demand measurements to detect any coordinated cyber assaults intended to manipulate the distribution of energy. Furthermore, anomaly detection [60,66,67] can be combined with secure authentication methods and protected communication channels to prevent unauthorized access arising from exposed credentials. These techniques establish a comprehensive defence framework that detects suspicious behaviour and imposes operational limitations, thereby protecting the integrity, dependability, and financial stability of EVCNs within complex smart grid systems.

**Table 2:** High-risk attacks in IoT and EV charging networks and their relevance for ML Security.

Attacks	Relevance for ML Security
OCPP exploits [63], grid manipulation [68], billing fraud	Detect anomalous charging patterns, load anomalies, fraud detection
Device hijacking, botnets, weak authentication [9,69]	Classification of network traffic, anomaly detection, botnet detection
Man-in-the-Middle (MitM) [70], replay [71], DoS [16], data leakage [9]	Sequence inconsistencies, traffic anomalies, intrusion detection [70]
Data injection/false data attacks [72]	Critical for ML integrity (poisoning, misleading analytics)
Insider misuse/credential compromise [64,73]	Behavioral anomaly detection (user profiling)
Traffic pattern anomalies	Time-series anomaly detection (ML-based IDS) [16,74]

Despite their success, traditional ML–centered security systems exhibit several fundamental limitations that hinder their effectiveness in dynamic, large-scale environments. One major challenge is their reliance on static training data. Conventional ML models are typically trained offline on historical datasets and deployed under the assumption that future data distributions remain stable. In real-world IoT and network environments, however, traffic patterns, user behaviour, and attack strategies evolve continuously, leading to data drift and concept drift [7]. When such drift occurs, model predictions become increasingly unreliable unless the system is retrained or adapted.

**Figure 1:**
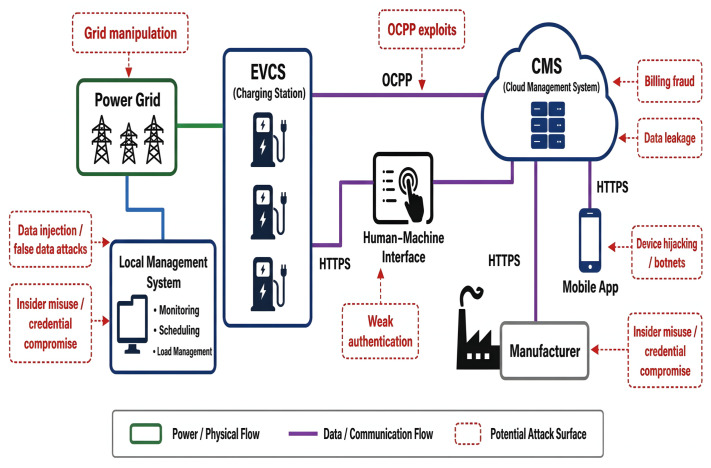
Potential attacks on EV infrastructure and protocols.

**Figure 2:**
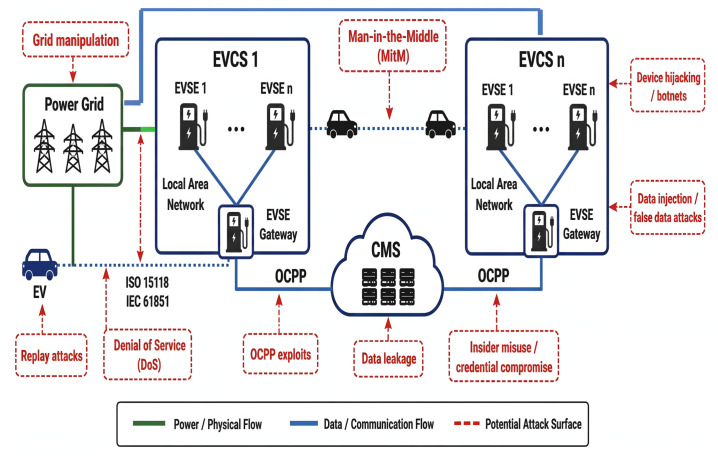
Potential attacks on EV ecosystem interactions.

**Figure 3:**
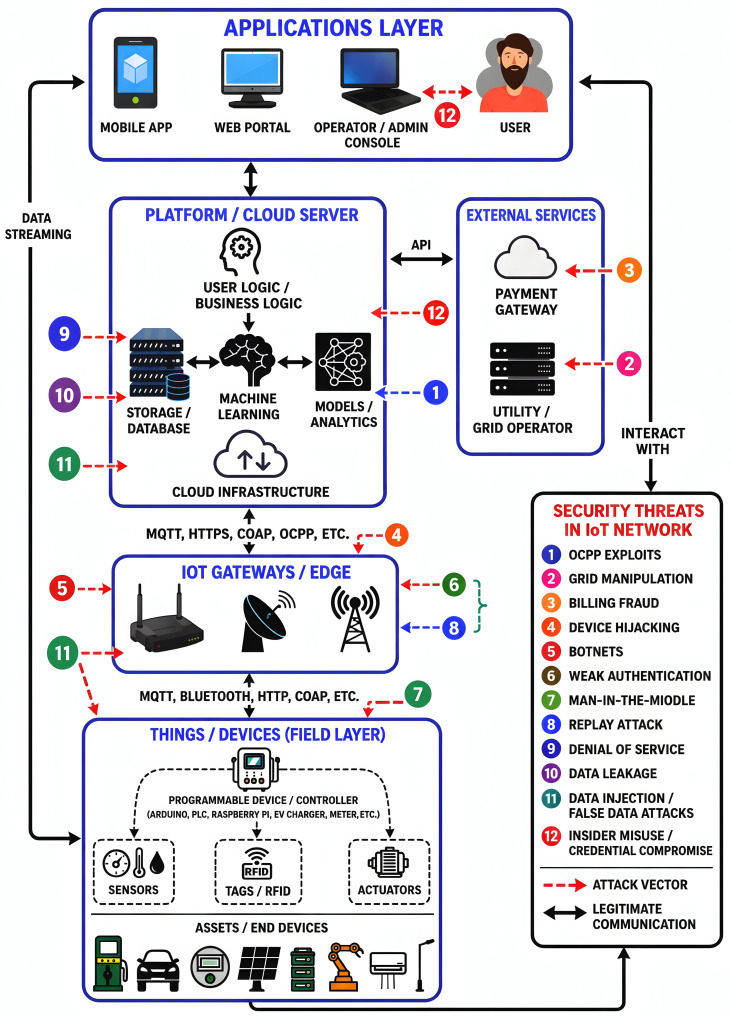
Potential attacks on IoT system building blocks.

We present the following diagrams that map attacks to IoT and EV charging networks, taking into account the cyber threats we discussed. Figure 1 and Figure 2 show potential attacks on EV infrastructure and protocols, as well as on EV ecosystem interactions. The red arrows indicate the locations within the EV charging network that are being targeted by different attacks. Figure 3 illustrates the potential attacks on the building blocks of IoT systems. The EV diagrams (Figure 1 and Figure 2) were adapted from reference [75], while the IoT diagram, Figure 3, was adapted from reference [76] and extended with potential attack vectors.

### 3.2. Challenges of Traditional ML-Based Security Measures

**Figure 4:**
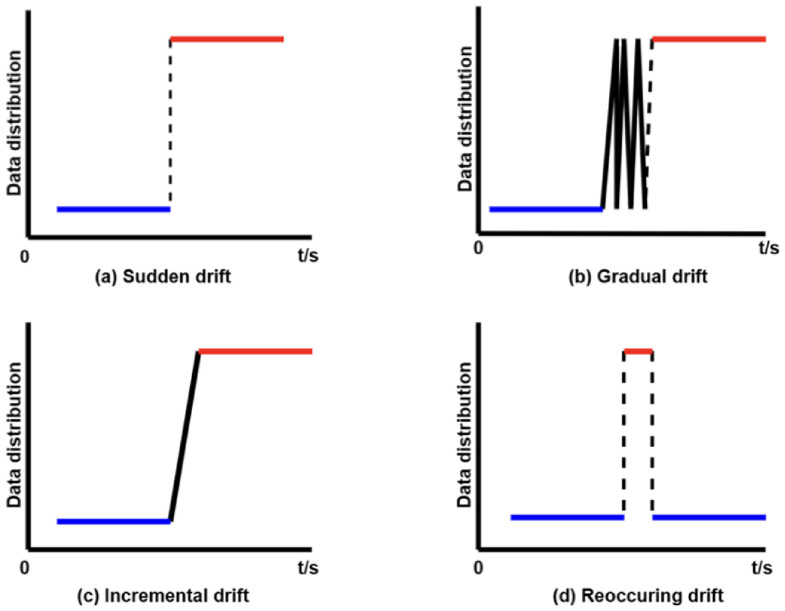
Types of drifts [79].

The IoT is characterized by a vast and continuously expanding network of interconnected devices that collect and transmit data in real time. This data is increasingly used to build intelligent services in domains such as smart grids, industrial automation, and environmental monitoring. However, effectively leveraging this data with ML introduces substantial challenges. Among the most significant challenges are heterogeneity, high dimensionality, and dynamic environments, which lead to concept drift and data drift [2,8]. Figure 4 shows a diagrammatic representation of different types of drifts. Figure 4a shows sudden drift, where the change occurs abruptly. Figure 4b shows gradual drift, where old and new concepts coexist during a transition period. Figure 4c incremental drift, where the concept changes continuously over time. Lastly, Figure 4d recurring (seasonal) drift, where previously observed concepts reappear after some interval. The blue line in Figure 4 represents the data before drift, while the red line represents the data after drift.

Heterogeneity in IoT data stems from variations in device types, communication protocols, data formats, and sampling rates, posing a significant challenge to the effectiveness of traditional ML pipelines. Traditional ML systems typically rely on predefined preprocessing, feature engineering, and model selection strategies, which often assume uniform data structures. As a result, heterogeneous inputs can lead to suboptimal feature representations, pipeline incompatibilities, or degraded model performance across different devices or contexts [2].

High dimensionality further complicates traditional ML operations. While AutoML frameworks aim to automate feature selection and model optimization, the presence of large, noisy, or redundant feature sets increases the search space for model architectures and hyperparameters. This not only increases computational cost but also increases the risk of overfitting, particularly when the framework lacks effective dimensionality reduction or regularization mechanisms. In practice, high-dimensional data can cause AutoML systems to favour overly complex models that generalize poorly in real-time deployments [2,8].

The ever-changing nature of IoT ecosystems, marked by concept and data drift, highlights the limitations of static AutoML pipelines. Concept drift, in which the relationship between features and labels changes over time, and data drift, in which the distribution of input features evolves, both require continuous model adaptation.

Model drift occurs when the spread of the training data diverges from that of the prediction data (real-world data). A clear indicator of model drift is the degradation of model performance over time, for example, a drop in accuracy for a supervised classification model [77]. Model drift can result from data or concept drift, both of which are significant concerns given the nature of IoT network traffic data. IoT data are dynamic streams from a highly interconnected, ever-changing, real-time IoT network environment. Therefore, machine learning on general IoT data is challenging, as data distributions and patterns evolve faster than new models can be developed to maintain good performance [8].

Data drift occurs when the distribution of input features shifts over time, causing the model to encounter input patterns not present in the original training set. Data drift can be caused by a range of real-world and system-level factors, including changes in user behaviour or demographics, alterations in data acquisition mechanisms (e.g., a faulty sensor in an IoT network), or modifications to data pipelines and preprocessing logic [78]. Because data drift alters the statistical properties of input features, it can create a gap between what the model learned during training and what it encounters during inference, even when the underlying prediction task remains the same.

Importantly, not all data drift immediately degrades model performance; if the drift occurs in a feature space that the model already generalizes well to or is less significant, performance may remain stable. However, when drift shifts the input distribution into sparser or error-prone regions of the important feature space, the model’s predictions may become unreliable [78]. Moreover, sustained or cumulative data drift can lead to concept drift, in which changes in input distributions alter the relationship between attributes and the target variable. In this case, the model’s learned decision boundaries no longer reflect the true mapping between inputs and outputs, effectively shifting the prediction problem itself.

The growth of the EV industry, as mentioned in Section 2, is a clear example of data/concept drift. Drift occurs in EV charging and IoT network environments when both attackers and the industry itself gradually change how network traffic appears without immediately changing the underlying objective. Intrusion-detection systems in EV infrastructure are typically trained on early datasets that contain clear anomalies, such as abnormal packet rates, malformed payloads, or irregular command sequences in OCPP communication, firmware update traffic, and smart meter telemetry. However, the rapid growth of the EV ecosystem, particularly large-scale urban fast-charging hubs, smart-charging algorithms, higher current levels, shorter charging sessions, and more frequent OCPP control messages. These evolutions alter the statistical distribution of normal traffic. Although these changes are legitimate, they introduce significantly higher traffic volumes and different communication patterns than those in earlier datasets, thereby gradually reducing IDS accuracy.

At the same time, attackers exploit this evolution by modifying how malicious traffic is represented at the protocol level rather than changing the attack goal. Instead of generating clearly abnormal behaviour, they embed malicious actions inside syntactically valid OCPP messages, replay legitimate firmware-update requests with slight modifications, or manipulate smart-meter data within realistic consumption ranges. From the model’s perspective, protocol-level features such as message timing, payload structure, session duration, transaction identifiers, and power-usage patterns begin to resemble legitimate traffic. This represents data drift, where the input feature distribution shifts while the classification objective remains unchanged.

If this process continues, concept drift eventually emerges. New technologies such as smart-charging coordination, load balancing, V2G communication, and automated firmware updates can generate traffic patterns that previously resembled attacks, such as rapid command sequences or frequent session interruptions.

Individually, these drifts may be minor and not immediately degrade model performance. However, collectively, they shift the relationship between attributes such as text patterns, sender metadata, traffic patterns, and the classification target. As a result, the model’s static decision boundary no longer aligns with the evolved input-output mapping, and what was once correctly classified as legitimate or abnormal may now be misclassified, leading to higher false positives. Such cumulative drift typically remains undetected until new ground-truth labels reveal the degradation, illustrating how incremental data shifts can trigger full concept drift. Additionally, concept drift can be classified into three types: gradual, abrupt/sudden, and incremental [79], each corresponding to the rate of change in the data. Most cybersecurity systems exhibit incremental concept drift, making it harder to detect anomalies over extended periods, often without noticeable statistical differences between consecutive instances [80].

When concept drift and data drift are addressed manually, traditional ML-based security systems require significant human effort, rely on specialized expertise, and deliver suboptimal model performance. Another major limitation is the heavy dependence on human intervention: conventional ML pipelines require extensive manual work for feature engineering, model selection, hyperparameter tuning, and periodic retraining. These processes are time-consuming, error-prone, and highly dependent on domain knowledge, making them difficult to scale and maintain in complex and dynamic security environments. Additionally, suboptimal design decisions can degrade detection performance, increase false positive rates, and reduce generalization to novel threats.

Moreover, fixed model architectures and manually tuned configurations often fail to maintain optimal performance across heterogeneous datasets and evolving deployment contexts. As network conditions and attack patterns shift, models that were previously effective may quickly become outdated, yet identifying and deploying superior alternatives typically requires substantial human intervention. This lack of autonomous adaptability motivates the transition from conventional ML security pipelines to Online AutoML, which can support continuous adaptation, automated model selection, and more resilient deployment under dynamic conditions.

### 3.3. Summary of Research Gaps and Our Contribution

Given the nature of cybersecurity challenges in IoT and EV ecosystems, existing protection mechanisms have largely evolved along separate, uncoordinated research trajectories. Conventional rule-based and signature-dependent approaches remain effective only for previously observed threats and have limited capability to handle adaptive or evolving attack behaviours. Machine learning-based methods improve detection performance through anomaly recognition, behavioural analysis, and classification; however, they are largely developed as static, domain-specific solutions trained offline under fixed datasets and assumptions, which limit operational adaptability in dynamic environments.

In parallel, research in online learning focuses on adapting models to non-stationary data streams, while AutoML and automated machine learning (AML) aim to reduce manual intervention through automated model selection and hyperparameter optimization. Despite their individual strengths, these paradigms remain largely disconnected, often applied independently rather than as a unified framework. Consequently, current approaches suffer from a structural limitation: they lack an integrated, continuously operating, and autonomously maintained cybersecurity framework that simultaneously supports real-time learning, adaptive model evolution, and automated optimization in dynamic IoT-enabled EV environments.

This paper addresses this gap by consolidating and bridging the previously fragmented research domains of IoT cybersecurity, EV charging security, online learning, and AutoML toward a unified framework for online, self-adaptive, and autonomously maintained ML-centered security systems.

**Figure 5:**
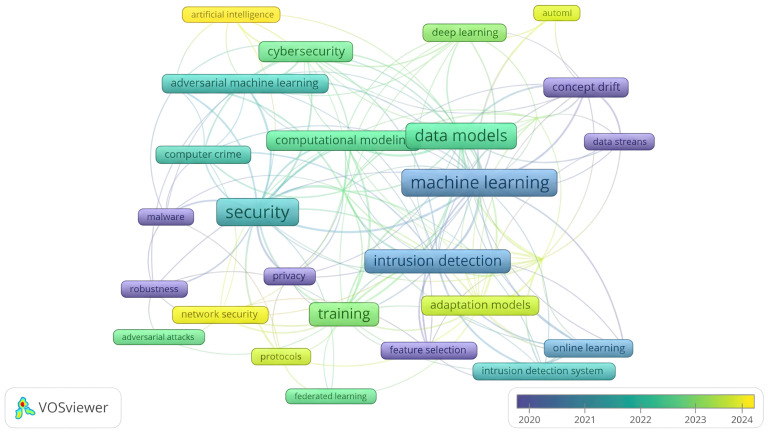
Overlay visualization of keyword co-occurrence.

A bibliometric co-occurrence/keywords analysis was performed using VOSviewer 1.6.20 to better examine the research ecosystem and topic links within the reviewed articles. Author keywords were derived from the selected articles and were examined to identify prevailing research themes, emerging trends, and interrelations among topics related to AutoML, online learning, cybersecurity, IoT, and EV charging networks. The resulting graphical representation (Figure 5) organizes keywords by co-occurrence frequency, with node size reflecting keyword significance, link strength representing the degree of association among keywords, and colours indicating thematic categories across the research area. This analysis offers a visual depiction of the conceptual framework of the domain and emphasizes the increasing convergence of adversarial ML, IDS, drift adaptation, and smart transportation security within contemporary AI-powered cyber–physical infrastructures.

## 4. Online AutoML

The primary motivation for AutoML is to enhance the accessibility, efficiency, and scalability of high-performance machine learning by minimizing the need for expert involvement. AutoML seeks to automate the lengthy, time-consuming, and complex tasks of building, tuning, and deploying ML models, thereby lowering the barrier to entry for non-experts [5,81]. This interest is particularly significant given the gap between the rapidly increasing demand for ML/AI applications in research and industry and the limited number of available experienced data scientists and ML engineers [82,83].

To bridge this gap, AutoML systems aim to automate tasks that typically require deep technical knowledge and substantial manual effort, including algorithm selection, hyperparameter optimization, feature preprocessing, and pipeline composition [5,84]. By packaging these steps into automated pipelines, AutoML democratizes access to ML, enabling domain experts in business analytics, healthcare, industrial IoT, and finance to build and deploy ML solutions with minimal statistical or programming experience [83,85].

Beyond accessibility, AutoML also delivers significant productivity and reliability gains. It reduces time-consuming, error-prone experimentation, accelerates prototyping and deployment cycles, and facilitates scalable ML solutions across diverse application domains [81,86]. Moreover, AutoML frameworks often leverage advanced optimization methods, such as meta-learning, ensembling, and neural architecture search (NAS), which can generate models that are comparable to, or even more efficient than, those designed manually by experts [82,84].

In practical settings, AutoML systems thus have a dual role: bridging the expertise gap for non-experts and augmenting the efficiency, robustness, and reproducibility of ML pipelines for expert practitioners. This dual role accounts for why AutoML is both a pragmatic necessity for powering AI adoption at scale and a research frontier in intelligent system automation.

In this section, we provide a comprehensive overview of Online AutoML, covering the fundamentals of AutoML and online learning and summarizing existing surveys. Additionally, an end-to-end conceptual architecture highlighting the core components of Online AutoML for cybersecurity is presented in Section 4. This progression is important because Online AutoML builds directly on the foundations of conventional AutoML while extending them to streaming, non-stationary, and resource-constrained environments.

### 4.1. Defining AutoML

AutoML is the process of automating the end-to-end pipeline for developing machine learning models [5,82,84]. AutoML has been defined in various ways as a solution that reduces dependence on data scientists by enabling domain specialists/experts to build ML applications with limited ML expertise [5]. Similarly, it can be broadly defined as the integration of automation with machine learning to optimize both time and resources, including human and computational resources. In more informal descriptions found in other papers and articles, AutoML is often described as a system that attempts numerous combinations of algorithms, hyperparameters, and preprocessing steps, thereby alleviating the need for manual intervention [87]. These informal definitions emphasize ease of use and aim to free professionals from trial-and-error experimentation. However, they do not always highlight the specific optimization problem that AutoML systems are addressing.

To clarify the concept, researchers often define AutoML as an optimization problem, most commonly the Combined Algorithm Selection and Hyperparameter Optimization (CASH) problem [66,88]. Other optimization algorithms include ParamILS, Sequential Model-based Algorithm Configuration (SMAC), Gender-based Genetic Algorithm (GGA), and Iterated Race (Irace) [88]. The CASH problem provides a mathematical framework for understanding what AutoML does in the black-box setting. The idea is that a user has a set of machine learning algorithms, each with its own set of hyperparameters. For example, support vector machines have parameters such as kernel type and penalty parameter, whereas decision trees have parameters such as depth and the minimum number of samples per split. The challenge is to select not only the most effective algorithm but also the optimal set of hyperparameters for that algorithm simultaneously.

Formally, CASH problem is defined as [66,88]:

(1) $$\left(A^{*},\lambda^{*}\right)=\underset{A^{\left(j\right)}\in A,\;\lambda \in \Lambda^{\left(j\right)}}{arg min}\;\frac{1}{K}\sum_{i=1}^{K}L\left(A_{\lambda }^{\left(j\right)},\;D_{\mathrm{train}}^{\left(i\right)},\;D_{\mathrm{valid}}^{\left(i\right)}\right)$$

where A represents the set of candidate algorithms, each with its corresponding hyperparameter space $\Lambda^{\left(j\right)}$. The goal is to identify both the algorithm $A^{*}$ and the hyperparameters $\lambda^{*}$ that minimize the validation loss over *K* cross-validation folds.

This formulation shows that algorithm selection and hyperparameter tuning can be viewed as a joint optimization problem. For instance, an algorithm may appear weak with poor default hyperparameters; however, it can outperform others once tuned correctly. By combining the two into a single optimization problem, CASH captures the true search space of AutoML.

Therefore, the informal definition of AutoML is closely aligned with the CASH formulation. The informal definitions highlight convenience and usability, while the CASH formulation provides a rigorous mathematical foundation. Modern AutoML frameworks such as Auto-WEKA [88], auto-sklearn [89], and Microsoft’s Azure AutoML explicitly adopt the CASH perspective in their design.

Notably, CASH primarily addresses the model selection and hyperparameter optimization stages of the AutoML pipeline and, by itself, does not encompass full end-to-end automation [88]. As a result, additional components of the machine learning workflow have been targeted through complementary automation paradigms [90].

Automated Data Preprocessing (AutoDP) focuses on optimizing data preparation steps that strongly influence downstream model performance, including missing-value imputation techniques (i.e., median, mean, kNN-based, or model-based imputation) [91], feature scaling and normalization methods, categorical encoding schemes (e.g., one-hot, target, or ordinal encoding), outlier detection and treatment, and class imbalance handling through resampling or re-weighting. These preprocessing choices are often interdependent and dataset-specific, making manual selection both error-prone and susceptible to human bias [90].

Automated Feature Engineering (AutoFE) addresses the automated construction, transformation, and selection of features to improve representational quality [92]. AutoFE techniques include feature generation via mathematical transformations (e.g., logarithmic, polynomial, or interaction terms), aggregation and window-based features for temporal data, embedding-based representations for high-dimensional inputs, and feature selection mechanisms based on statistical tests, mutual information, regularization, or model-driven importance measures. In addition, AutoFE may incorporate representation-learning approaches, such as autoencoders or deep feature extractors, to learn latent structures in complex data [89].

Similar to CASH, both AutoDP and AutoFE can be formulated as optimization problems over large, hierarchical search spaces and addressed using comparable strategies, including Bayesian optimization, evolutionary algorithms, reinforcement learning, and meta-learning. By extending these optimization paradigms beyond model selection and hyperparameter tuning, AutoML frameworks move toward fully automated, end-to-end pipelines in which preprocessing, feature engineering, and modelling decisions are jointly optimized rather than treated as isolated stages [89,93].

### 4.2. Current AutoML Studies

A mature and fully developed AutoML system integrates multiple optimization techniques and algorithms, such as hyperparameter optimization (HPO), neural architecture search (NAS) for deep learning, ensembling, and meta-learning into a dynamic, end-to-end ML pipeline designed to autonomously create an optimized model with limited or no human interference [1].

Such mature and general AutoML frameworks are available on commercial platforms such as Google’s Vertex AI AutoML, Amazon’s SageMaker Autopilot, and Microsoft Azure AutoML, which offer Platform-as-a-Service (PaaS) solutions that enable users with limited machine learning expertise to build customized, high-quality models through graphical interfaces and minimal coding [6]. While these services improve accessibility, cloud-based AutoML services often sacrifice flexibility for convenience. While they streamline common workflows and abstract infrastructure complexity, they may restrict fine-grained hyperparameter configuration, algorithm selection, or custom preprocessing pipelines—features that are critical in domain-specific or niche applications. Studies also highlight concerns about vendor lock-in, limited transparency, and constrained customization relative to open-source or self-hosted frameworks [26,94].

Building on both formal and informal definitions of AutoML, the survey by Baratchi, Wang, van Rijn, and Hoos provides an overview of the AutoML field. [84] It provides a recent and comprehensive overview of what AutoML is, the challenges it seeks to address, and identifies three fundamental components of AutoML systems:

1. Search space: the set of possible design choices, including algorithms, hyperparameters, and pipeline configurations.
2. Search strategy: the methodology employed to navigate the search space, typically through optimization algorithms that generate and evaluate candidate pipelines or hyperparameter settings.
3. Performance evaluation: the assessment of candidate solutions on data, using techniques such as cross-validation or hold-out validation, to determine their generalization performance and relative effectiveness.

As noted in [84], although CASH is a core problem, many AutoML systems do not fully automate tasks beyond model building; that is, data preprocessing, feature engineering, and deployment are often only partially automated or left to human design. Thus, the actual practice of AutoML, as surveyed, may approximate CASH in certain aspects; however, in others, it is less generalizable. Additionally, some AutoML systems constrain the pipeline structure by using fixed templates rather than searching over arbitrary pipelines.

Several initial surveys have informed our understanding of AutoML, ranging from introductory overviews to domain-specific studies. Notable contributions include the general overviews provided by Hutter et al. [5] and Zöller and Huber [26], Karmaker et al.’s [82] first overall synthesis, Barbudo et al.’s [81] longitudinal review of AutoML trends, Baratchi et al.’s [84] formal and future framework, and IoT-based surveys by Yang and Shami [8,27]. They all contribute their individual perspectives to the evolving AutoML paradigm.

Hutter, Kotthoff, and Vanschoren’s pioneering survey [5] is among the first comprehensive surveys of AutoML systems. It addresses foundational components, including HPO, NAS, and meta-learning, as well as benchmark frameworks and standardized test datasets. The survey, nonetheless, focuses primarily on batch learning in static environments and omits crucial aspects such as data stream processing and concept drift, which are critical for real-time processing in IoT and edge computing.

Karmaker et al. [82] provide one of the earliest comprehensive syntheses of AutoML frameworks, positioning their contribution at a time when the field was beginning to move beyond academic proof-of-concept studies. A notable aspect of their survey is its examination of both open-source libraries, such as Auto-WEKA, Auto-sklearn, and TPOT, and commercial tools, such as Google AutoML, and its assessment of the limitations of these systems in real-world settings. The paper’s main contribution is to contextualize AutoML with respect to major challenges and opportunities, including scalability, interpretability, and computational cost. This establishes a research agenda that subsequent surveys have extrapolated to deep learning, reinforcement learning, and resource-constrained applications.

Similarly, Zöller and Huber [26] conduct a systematic benchmarking of standard AutoML frameworks with respect to usability, performance, and extensibility. While highly valuable for practitioners, the survey relies on typical ML pipelines and does not address issues specific to streaming data, adaptive learning, or edge deployment in IoT settings.

Filling this gap, Yang and Shami [8] present a domain-specific overview at the intersection of AutoML and IoT. Their work highlights significant challenges, including non-stationary data distributions, limited computational resources, and the need for real-time analysis in edge environments. They present an architectural framework that can be structured, as it comprises streaming analytics, concept drift detection, and resource-limited model adaptation, thereby positioning AutoML as a practical enabler of smart IoT systems.

With these as foundations, Barbudo et al. [81] offer a retrospective analysis of eight years of research on AutoML. The novelty of their work is the introduction of a categorization scheme that classifies AutoML approaches by their goals, optimization strategies, and application domains. What differentiates this effort from previous surveys, which largely focused on methods and systems, is this research’s emphasis on longitudinal trends, such as the emergence of deep learning-based AutoML and the increasing frequency of NAS. The main contribution is a comprehensive mapping of the field’s history, collating facts on search strategies, pipeline configurations, and meta-learning, and further identifying current gaps in scalability, fairness, and interpretability, thereby proposing future research directions.

The most recent and forward-looking synthesis was conducted by Baratchi et al. [84], integrating past developments with current capabilities and situating AutoML within the context of formal foundations. Their originality is two-fold: first, they attribute the intellectual roots of AutoML to John R. Rice’s algorithm selection problem and explicitly present the CASH problem as the basis of modern AutoML; second, they extend the survey to dynamic, online, and resource-constrained environments. Its main contribution is a comprehensive taxonomy that integrates conventional AutoML methods with emerging approaches, including meta-learning, ensembling, and NAS, and provides a research roadmap for future work. By identifying open challenges in robustness, transparency, scalability, and fairness, this work positions AutoML as both an engineering technology for democratizing machine learning and a research frontier central to future AI automation.

An earlier publication by the same authors [27] presents a lightweight, adaptive system (OASW) for IoT data streams. While not a traditional survey, it demonstrates how AutoML techniques—specifically, concept drift detection and online learning—can be operationalized using a continuous LightGBM-based approach. This work provides empirical validation on real-world datasets, demonstrating that AutoML can effectively address data drift in constrained-resource settings. Together, these publications provide both theoretical grounding and practical strategies for AutoML-driven analytics in dynamic, IoT-specific environments. However, they also show that streaming adaptation, explicit drift handling, and real-time operation remain less fully integrated in much of the broader AutoML literature. This gap directly motivates the move toward Online AutoML in IoT and EV charging environments.

### 4.3. Role of Online Learning in AutoML for IoT

Perhaps most critically, the dynamic nature of IoT environments, characterized by concept and data drift, highlights the limitations of static AutoML pipelines. Concept drift, in which the relationship between features and labels changes over time, and data drift, in which the distribution of input features evolves, both require continuous model adaptation.

Traditional batch-mode AutoML frameworks assume stationary data and a fixed training-validation split, which makes them ill-suited to streaming or non-stationary contexts. Once deployed, these models can become obsolete as they fail to detect and adapt to new patterns in the data [8]. Without an online learning mechanism, the entire pipeline—from preprocessing to model selection—may become outdated, necessitating frequent manual retraining or redevelopment, which defeats the purpose of automation. Therefore, addressing these challenges requires integrating online learning into AutoML systems to enable dynamic pipeline updates and real-time model adaptation [3].

Traditional ML and batch-mode AutoML frameworks, such as TPOT and Auto-sklearn, assume a static data distribution and require retraining on a full dataset to update models. In IoT environments, this paradigm is inadequate. Batch retraining is resource-intensive, inflexible, and vulnerable to outdated assumptions about the data. As such, these frameworks are rapidly becoming obsolete for real-time, high-frequency IoT applications [8].

To overcome these limitations, online learning has emerged as a critical component of modern AutoML systems for IoT, enabling models to update incrementally with each new data instance. Online learning enables continuous model adaptation, facilitating real-time response to data drift and improving robustness in evolving environments. This approach also supports low-latency operation, making it ideal for intrusion detection, anomaly detection, and predictive maintenance systems [3].

#### 4.3.1. Consequences of Concept/Data Drift

Data and concept drift have critical implications for both traditional and online learning systems when left unaddressed. Drift can cause a gradual degradation in model performance that can severely impact downstream applications, erode trust in model outputs, and introduce ethical and regulatory consequences. Without effective drift detection and countermeasures, machine learning systems are vulnerable to making outdated or harmful decisions, especially in high-risk or real-time environments.

In streaming and online machine learning environments, concept drift and data drift are even more crucial as the model learns and adapts from a continuous stream of data. They occur when the statistical properties of the input data or the underlying relationship between features and target variables change over time. If not managed effectively, such drift can degrade model performance and reliability, with serious consequences.

Reduced Model Accuracy and Reliability: The most immediate impact of concept and data drift is the deterioration in prediction accuracy. As models become misaligned with the evolving data distribution, their ability to generalize diminishes significantly. This can result in outdated models continuing to make poor predictions, particularly in time-sensitive applications such as fraud detection, industrial quality control, or cyber intrusion detection. Ref. [95] emphasizes that model drift is a key threat in data-stream environments, necessitating systems that can adapt dynamically to changes.

Increased Risk of False Predictions: With drift, models often produce higher rates of false positives and false negatives. For example, a security model trained on past network behaviours may incorrectly flag legitimate activities or overlook new attack patterns. These erroneous predictions can delay the detection of real anomalies and erode confidence in the system [7,8].

Operational and Economic Costs: In environments such as IIoT, where data-driven systems control real-time processes, drift can lead to delayed or inaccurate decisions, resulting in operational failures, economic losses, or even safety hazards. The inability to respond quickly to evolving data conditions can increase downtime and maintenance costs, or lead to failures of business-critical automation [8,22].

Frequent Manual Intervention: Without automated drift handling, teams often need to retrain or manually tune models. This reduces the scalability and practicality of deploying machine learning systems in production, especially in dynamic environments. As Celik et al. note, a lack of adaptive capacity in traditional models increases human workload and errors, thereby hindering real-time responsiveness [22].

Compromised Trust in Automation: Repeated model failures caused by model drift can erode user trust in AI-driven systems. Whether the end users are engineers, security analysts, or business decision-makers, visible inconsistencies in model performance can lead to reluctance to adopt or continue using automated solutions. Imrus et al. highlight that feature selection and model tuning alone are insufficient if the model cannot adapt to non-stationary data streams [87].

Regulatory and Ethical Implications: In domains such as healthcare, finance, and critical infrastructure, poor model performance due to unaddressed drift can also have regulatory implications. Models that become biased toward state-of-the-art (SOTA) methods or fail to comply with fairness and transparency standards may violate privacy laws or industry regulations [6].

#### 4.3.2. Concept and Data Drift in AutoML Models

Current offline AutoML approaches have limitations. One well-known constraint is their high computational demand; searching over large configuration spaces of models and hyperparameters is time- and resource-intensive. Additionally, the complexity of the resulting pipelines often reduces transparency, making them difficult to interpret or trust, particularly in critical applications. AutoML frameworks also face challenges with flexibility, often requiring structured tabular data and struggling with unstructured or highly customized inputs. Many AutoML systems assume stationary, batch datasets and are ill-equipped to handle streaming data or real-time requirements, which significantly limits their utility in dynamic environments [22,23] point out.

These limitations are particularly evident in dynamic or non-stationary environments, as in IDSs. In such settings, AutoML systems must contend with concept drift, where the data distribution changes over time and must adapt accordingly to maintain performance. Most traditional AutoML frameworks are not designed for this, as they train models offline and do not update them in response to new data. The novel AutoML framework ChaCha [23] addresses this gap by introducing mechanisms for continuous adaptation and faster response times. Similarly, the Online AutoML framework by [22] demonstrates adaptive model selection and hyperparameter tuning over data streams, thereby enabling lifelong learning capabilities in AutoML.

Current AutoML frameworks have proven particularly effective in addressing the multifaceted challenges associated with IoT data; namely heterogeneity, high dimensionality, and dynamic, drifting environments. Firstly, IoT systems often generate data streams from diverse sensors in varying formats and temporal resolutions, rendering manual pipeline design impractical and error-prone. AutoML alleviates this by automating feature engineering, algorithm selection, and hyperparameter optimization, thereby ensuring robust performance across heterogeneous tasks without extensive domain expertise [95]. Secondly, many IoT applications involve hundreds of sensor modalities, resulting in high-dimensional data that can lead to overfitting and scalability issues. AutoML frameworks often incorporate dimensionality reduction (e.g., LASSO, PCA) and feature selection methods to mitigate these risks while maintaining model interpretability and computational efficiency [87].

The most critical challenge for using AutoML in IoT, however, lies in concept and data drift, which refers to temporal changes in feature distributions and target functions that degrade the performance of static models [7]. In response, recent research has developed Online AutoML approaches that detect drift and dynamically adapt pipelines. Celik et al. [22] introduced OAML. This Online AutoML framework continuously reconfigures preprocessing, modelling, and ensembling components in response to evolving data streams, showing superior adaptability compared to conventional online learners. Similarly, Yang and Shami [4] demonstrated that embedding a drift-aware sliding-window strategy within a LightGBM pipeline enabled real-time adaptation for anomaly detection in IoT streams. ARCUS [96] further exemplifies state-of-the-art methodology, employing an adaptive pool of deep autoencoders alongside drift-driven model updates to maintain accuracy on evolving high-dimensional streams.

Online AutoML extends traditional AutoML by incorporating online learning algorithms, drift detectors, and stream-based hyperparameter tuning. Compared to batch AutoML, which treats the learning problem as static, Online AutoML continuously optimizes the model pipeline as it processes incoming data streams. However, this flexibility introduces new complexities: model parameters become sensitive to the rate and magnitude of concept drift. For example, if drift is abrupt, online models may overfit to transient anomalies unless equipped with adaptive mechanisms such as sliding windows, decay factors, or ensemble techniques [27].

In sum, the evolving nature of IoT data necessitates adaptive ML pipelines that respond to concept drift and heterogeneity in real time. Online learning is not merely an enhancement but a foundational requirement for AutoML systems that operate in dynamic IoT/EV environments. However, in the next section, the benefits of Online AutoML are discussed.

## 5. Online AutoML Architecture

As outlined in foundational works such as [5,97], the core components of AutoML include automated data preprocessing and feature engineering, model selection from a diverse portfolio, HPO, ensembling to enhance performance, and meta-learning to transfer knowledge across tasks. Current AutoML components orchestrate the delivery of high-performing machine learning models with minimal human intervention, while integrated evaluation mechanisms ensure robust performance estimation.

Building on existing AutoML components, Online AutoML extends AutoML for dynamic environments where data arrive continuously and drift over time. Unlike traditional AutoML systems designed for static datasets, Online AutoML frameworks must handle real-time learning under constraints such as limited memory, latency, and concept drift [22,23,95]. To our knowledge, most existing Online AutoML architectures adopt either a monolithic design or a modular pipeline structure, facilitating real-time learning and reconfiguration [22,96]. For example, the ChaCha framework employs a caching-based bandit approach to manage pipeline decisions efficiently [23]. Whereas OAML [22] conceptualizes Online AutoML as a sequential interaction between an AutoML module and an online learning module. This enables OAML to organize components as decoupled units, facilitating plug-and-play configuration and component-level retraining [22]. However, these exist. Online AutoML architecture processes are designed in segregation, which limits adaptability because each stage operates with limited awareness of the evolving data stream.

**Figure 6:**
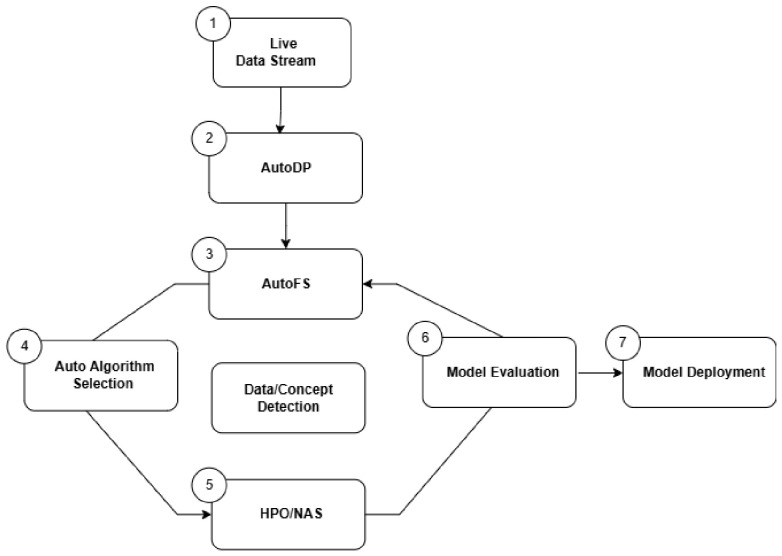
Conceptual diagram for Online AutoML.

In contrast, the proposed cyclic Online AutoML framework comprises specialized components tailored for streaming scenarios: online data preprocessing (AutoDP), incremental feature engineering (AutoFS), model selection with lightweight HPO/NAS optimization techniques, concept/data drift detection and adaptation, and continual performance evaluation (Figure 6). While most of the above components are also found in conventional AutoML, how they are used in an online and dynamic environment will be discussed in the following sections. However, a key feature of the proposed Online AutoML architecture is the incorporation of a feedback mechanism that detects both data drift and concept drift. When such changes in data and/or performance are detected, the framework automatically re-initiates earlier stages of the pipeline, including feature engineering, algorithm selection, and hyperparameter optimization. This feedback mechanism ensures the models remain accurate, adaptive, and efficient in real-world applications characterized by non-stationary data and operational constraints.

It is critical to note that the architectural design of an Online AutoML system is not merely an implementation concern but a fundamental determinant of whether the system can effectively support continuous data stream processing and adaptive model evolution under real-world constraints. Many existing Online AutoML approaches implicitly assume that model adaptation can be performed sequentially, without explicitly accounting for the associated computational overhead, latency implications, and resource trade-offs. As a result, the architectural choice becomes a central research challenge, as it must simultaneously ensure scalability, low-latency response, and the structural flexibility required to incorporate evolving components such as new learning algorithms, optimization strategies, or drift-detection mechanisms. This issue is particularly pronounced in high-velocity, resource-constrained environments, including Industrial Internet of Things (IIoT) systems and cybersecurity applications, where rapid changes in data distributions and strict real-time requirements expose the limitations of conventional AutoML architectures [2,8].

### 5.1. Automated Online Data Pre-Processing AutoDP

In streaming environments, data preprocessing is a crucial first step that handles missing values, performs categorical encoding, normalizes data, and filters noise in real time. Unlike batch settings, preprocessing in Online AutoML must work incrementally to minimize memory overhead and latency. Techniques like online normalization using rolling statistics and incremental one-hot encoding are commonly employed [8,87]. For instance, the ARCUS framework integrates adaptive normalization strategies to maintain feature scale consistency in high-dimensional streams [96]. Proper preprocessing improves the quality of downstream learning and guards against input drift, which can otherwise degrade performance unpredictably over time [7].

Preprocessing is a fundamental stage in machine learning pipelines, directly determining the quality and reliability of model outputs [98,99,100,101]. Its primary role is to transform raw, noisy, or incomplete data into consistent and informative representations that maximize the learning capability of algorithms [102,103,104]. In AutoML pipelines, preprocessing is particularly critical because it lays the foundation for model selection, hyperparameter optimization, and incremental learning [105,106]. Without effective preprocessing, even the most sophisticated algorithms cannot compensate for poor-quality inputs, leading to unreliable or biased predictions [107,108].

Preprocessing directly impacts model performance by ensuring data quality and consistency [98,99]. Proper cleaning, scaling, encoding, and balancing help models focus on meaningful patterns, improve robustness against noise and missing values, reduce overfitting, and enhance generalization [102,103]. Well-prepared data also improves training efficiency and reduces computational costs, which are essential for real-time IoT applications [101,109]. Addressing class imbalance, for example, through SMOTE or adaptive oversampling, ensures that rare but essential events, such as intrusions, in IoT datasets are reliably detected [98,106,110].

**Figure 7:**
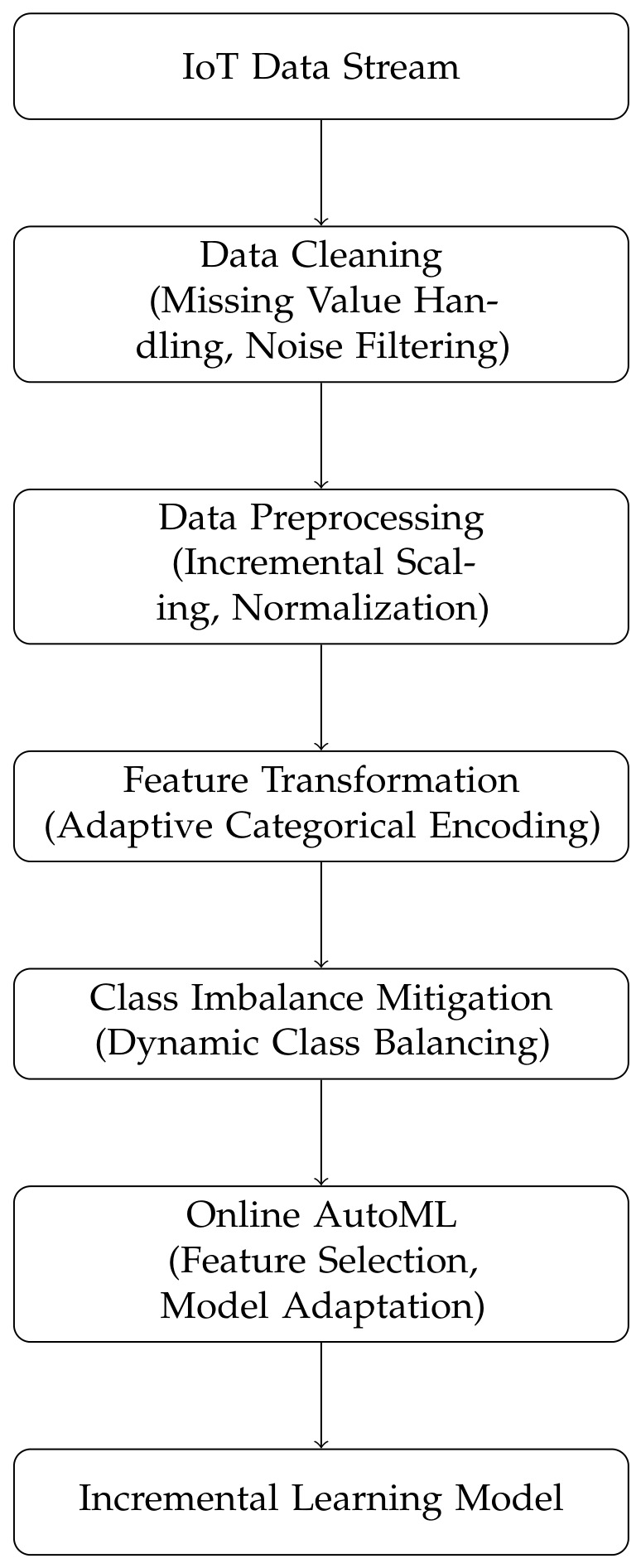
Online learning pipeline for streaming IoT data.

An ideal online preprocessing pipeline for live IoT IDS data may follow the sequence as shown in Figure 7.

This design ensures that data streams are continuously transformed into clean, reliable, and model-ready representations, enabling adaptive and accurate intrusion detection.

Preprocessing is a foundational determinant of AutoML performance in IoT IDS environments. Static preprocessing pipelines are insufficient due to the dynamic, noisy, and imbalanced nature of IoT data. Incorporating incremental scaling, adaptive encoding, online imputation, and dynamic class balancing allows AutoML frameworks to maintain high-quality inputs, improve model robustness, and detect rare events in real time [98,99,108,110]. Future research should focus on fully integrating these automated and adaptive preprocessing components into Online AutoML systems for IoT security applications.

Additionally, online automated preprocessing, also known as online automated data preprocessing, cannot be treated as a static, one-time operation. When concept drift, data distribution shift, or pipeline-level changes (e.g., model replacement, hyperparameter re-optimization, or ensemble reconfiguration) are detected, previously learned preprocessing parameters may become invalid. Under such conditions, reprocessing of incoming data is essential to maintain consistency between the data representation and the active model configuration. For example, changes in feature distributions necessitate recalibrating normalization statistics, whereas the introduction of a new model may require alternative encoding schemes or feature transformations. Failure to realign preprocessing with model updates can result in feature mismatch, degraded performance, or unstable learning dynamics, particularly in streaming intrusion detection scenarios where data distributions evolve rapidly [7].

Accordingly, advanced Online AutoML pipelines must incorporate drift-aware and model-aware preprocessing, in which preprocessing parameters are continuously monitored and selectively reset, adapted, or relearned in response to detected changes. This includes recomputing rolling statistics, updating categorical encoding, revisiting feature relevance, and re-balancing class distributions after drift events or pipeline reconfiguration. Such tight coupling between preprocessing and automated model adaptation is a defining requirement for truly autonomous Online AutoML systems and distinguishes them from conventional adaptive learning pipelines.

#### Limitations of Static Data Preprocessing Pipelines

Most traditional frameworks implement preprocessing as a static, offline step, assuming fixed data distributions and batch availability [99,105]. Static pipelines are limited in dynamic IoT environments, which feature non-stationary, noisy, and incomplete data streams [101,104]. Fixed normalization and class-balancing methods cannot adapt to data and concept drift, thereby reducing model performance and reliability. Consequently, online or incremental preprocessing strategies are necessary for effective IoT intrusion detection.

Recent studies demonstrate several preprocessing techniques which can be adapted for online or incremental execution [98,108,110]: These algorithms are available to the public via Python libraries such as scikit-learn, TensorFlow/Keras, and PyTorch.

- Incremental Scaling and Normalization: Online Z-score or Min–Max scaling allows models to handle shifting numeric distributions without retraining [102,111].
- Adaptive Categorical Encoding: Hashing-based or incremental one-hot encoding enables dynamic handling of new categories in streaming data [109,112].
- Online Imputation and Outlier Management: Sliding-window or incremental imputation methods, along with streaming outlier detection, enhance robustness to missing or anomalous values [99,107].
- Dynamic Class Balancing: Adaptive oversampling or hybrid re-balancing ensures that minority classes, such as intrusions, remain represented as data streams evolve [98,110,113].
- Data Cleaning and Noise Filtering: Duplicate removal, de-noising, and outlier mitigation are applied incrementally to maintain high-quality input for online learning [98,113].

Although many studies claim online or real-time operation, only a limited number explicitly describe how data preprocessing is carried out under live-streaming conditions. The available literature indicates that preprocessing is often treated as a coarse-grained or implicit step, with little transparency regarding how it is adapted online or controlled within an AutoML pipeline.

This limitation is evident across several representative approaches. The AOC-IDS framework, for example, employs an incremental batch-based strategy in which the model is initially trained on 20% of the dataset and subsequently updated with successive 1.6% increments [61]. While this design supports online learning, preprocessing operations—such as normalization and categorical encoding—are assumed to remain fixed and are not described as adaptive or responsive to data drift. More explicit streaming architectures are observed in adaptive neural network systems, where live traffic from SPAN ports, TAP devices, and NetFlow agents is ingested through platforms such as Kafka and Flink or Spark Streaming, aggregated into microsessions, and normalized into feature vectors before learning [114]. Nevertheless, these works do not elaborate on how preprocessing parameters are updated when the data distribution or model configuration changes.

Other studies focus on data filtering as an online preprocessing step. For instance, the OI-SVDD with AS-ELM framework applies rate-of-convergence-based filtering to avoid data saturation and stabilize incremental learning [115]. While effective for controlling stream dynamics, this filtering operates independently of broader AutoML decisions and does not extend to automated feature transformation or encoding adaptation. Similarly, MLOps-integrated intrusion detection frameworks incorporate automated drift detection and retraining triggered by metrics such as the Population Stability Index [116]. Yet, they provide limited insight into how preprocessing parameters are recalibrated after drift detection.

Overall, existing systems tend to restrict online preprocessing to basic operations such as aggregation, normalization, filtering, or batch-wise handling, rather than implementing adaptive, AutoML-driven preprocessing pipelines. Offline AutoML and NAS-based approaches, including AutoMHS-GPT and DDOSNAS, further confine preprocessing to the training phase and do not support reprocessing during live deployment or post-deployment pipeline evolution.

### 5.2. Drift Detection and Adaptation Mechanisms

As noted in earlier sections, the defining challenge in Online AutoML is data/concept drift, in which the data distribution changes over time, violating the assumption of stationarity. Drift detection modules monitor data and model outputs to detect such changes and trigger adaptation.

Concept drift represents a fundamental challenge in Online AutoML environments where the statistical properties of the target variable or the relationship between input features and outputs change over time, or a degradation of performance below a defined threshold violates the stationarity assumption upon which most machine learning models are built [7]. Concept drift directly degrades model performance, as models trained on historical data distributions become increasingly obsolete when faced with evolving patterns [7,95]. In domains such as IoT anomaly detection and cybersecurity, this deterioration can lead to inaccurate predictions that compromise system security and produce unreliable operational decisions [8,27]. Undetected drift creates a silent failure mode where models continue to operate with declining accuracy without alerting practitioners to the underlying problem. The longer the drift remains unaddressed, the more severe the model’s misalignment with current data patterns becomes, potentially rendering the model entirely ineffective [117].

To maintain a robust Online AutoML system, data and concept drifts must be systematically detected, and an appropriate adaptation mechanism is central [7,22]. The drift management process involves two distinct but interconnected phases: detection and adaptation [7]. Detection mechanisms continuously monitor either the data distribution or model performance to detect significant changes. Once drift is detected, the system must adapt through one or more strategies—such as incremental model updates, complete retraining, warm-started re-optimization, or pipeline restart—to restore predictive performance [22,95]. This detect-then-adapt paradigm ensures that computational resources are deployed efficiently, triggering expensive retraining operations only when statistically justified rather than on arbitrary schedules [22,27].

Modern drift detection and adaptation algorithms can be broadly categorized into two complementary approaches: explicit and implicit detection methods [7,27]. Explicit drift detection methods employ statistical tests or mathematical inequalities to monitor changes in data distributions or feature statistics directly [7]. These methods operate as “white-box” systems, providing interpretable signals about when and where drift occurs. Representative algorithms include Drift Detection Method (DDM), Early Drift Detection Method (EDDM), and Adaptive Windowing (ADWIN) [7]. DDM monitors the online error rate of a classifier, assuming, under PAC learning, that error decreases with more samples under stationary conditions, and defines warning and drift thresholds based on error-rate statistics [7]. EDDM improves upon DDM by tracking the average distance between consecutive classification errors, rather than only the error rate, making it more sensitive to gradual concept drift while maintaining good performance under abrupt changes [7]. ADWIN employs a fundamentally different approach by maintaining a variable-length sliding window that dynamically grows when no change is detected and shrinks when drift occurs, dividing the window into two sub-windows and comparing their statistical means using McDiarmid’s or Hoeffding’s inequality [7]. When the difference between sub-window averages exceeds a threshold defined by this inequality, drift is detected, and older data are discarded, thereby providing mathematical guarantees for false-positive and false-negative rates [7,27].

Effective drift detection is essential for maintaining robust learning in dynamic environments such as IoT and cybersecurity, where data distributions evolve continuously. Based on the systematic review, online AutoML frameworks employ multiple complementary mechanisms to detect drift. Most systems rely on data-driven monitoring, where models continuously track changes in incoming network traffic patterns. For example, the Mateen framework adapts to changing benign network behaviour, while VAE-based approaches monitor traffic distributions in real time [60,114]. In addition to data-driven detection, several frameworks implement statistical drift detection, such as the IDNet model, which uses the Population Stability Index (PSI) to identify distribution changes and automatically trigger retraining when predefined thresholds are exceeded [116]. Lastly, performance-based detection is widely adopted, in which adaptation is initiated when model accuracy begins to degrade, particularly in reinforcement-learning-based adaptive IDS frameworks [62].

Once drift is detected, the dominant response strategy is incremental learning, in which model parameters are updated continuously without full retraining. This approach enables models to adapt to evolving network behaviours and attack signatures while significantly reducing computational overhead [62]. Efficiency is further improved through representative sample selection, as demonstrated by the Mateen framework, which requires labelling only 1% of the incoming data while maintaining detection performance [60]. More advanced systems aim to eliminate human intervention. For instance, AOC-IDS implements autonomous pseudo-labelling to create a fully self-adaptive “labour-free online framework for continual adaptation” [61]. At the same time, VAE-based models perform real-time parameter updates using an online learning mechanism that adapts “on the fly” [114].

To address more complex drift scenarios, several frameworks incorporate specialized adaptation mechanisms. Hybrid IDS models employ dual-phase detection, combining ensemble-based classification for known threats with clustering-based outlier detection for unknown attack patterns [62]. Memory-driven frameworks such as Auto-CIDS further enhance adaptability by employing active learning with memory buffers, enabling models to leverage past experiences during adaptation [118]. Finally, to mitigate catastrophic forgetting, recent IIoT-focused frameworks adopt techniques such as Elastic Weight Consolidation, enabling models to adapt to new intrusion patterns while preserving previously learned knowledge [119].

These examples illustrate a clear evolution in Online AutoML for cybersecurity: from simple periodic retraining to a layered, intelligent approach. Modern systems integrate multi-faceted detection triggers with efficient, targeted adaptation responses, achieving varying levels of automation—from minimal human intervention (1% labelling) to fully autonomous operation. This sophisticated drift management is essential for maintaining robust, scalable, and sustainable defences in dynamic network environments.

### 5.3. Automated Online Feature Selection

Automated Online Feature Selection (AOFS) refers to techniques that automatically select, modify, or eliminate inputs as data are received in a continuous stream (batches or sample-by-sample) [120]. In contrast to traditional feature selection, which is performed once on a static, immutable dataset, AOFS must operate under the constraints imposed by streams of security data (e.g., concept drift, high throughput, large dimensionality, and limited computational resources on edge devices). AOFS systems independently determine which attributes to preserve for a given model and continuously adjust these selections without human intervention [120]. AOFS is a crucial concept in online learning models for several reasons.

First, contemporary networks, IoT devices, and endpoints generate numerous raw features (e.g., headers, flows, and payload-based signals). Continuously processing every characteristic is costly and latency-inefficient [121]. This computational burden is exacerbated by the “curse of dimensionality”, in which the volume of the data space grows exponentially with each additional feature, making statistical learning and precise density estimation from limited streaming samples increasingly challenging and resource-demanding [122]. In the absence of feature selection, the prevalence of numerous irrelevant and redundant attributes, which are typical in highly correlated network traffic data, has the potential to obscure significant patterns, induce model overfitting, and ultimately compromise the overall efficacy of the intrusion detection system [123,124]. Therefore, by reducing the dimensionality of the high-dimensional feature space in real time, AOFS mitigates runtime costs and memory usage. This efficiency is paramount for enabling the operation of advanced machine learning models on edge devices with stringent power and processing constraints [125].

Secondly, the signatures associated with cyberattacks and benign behavioural patterns evolve; characteristics previously deemed predictive may now represent noise. A static feature set selected during an initial training phase can rapidly become obsolete, failing to capture new attack vectors or updated normal behavioural patterns [126]. For instance, a feature that effectively identifies a particular exploit may lose its discriminative power if the attack methodology is altered or if network protocols are modified. Consequently, AOFS enables models to adapt to the shifting significance of features in real-time [120]. AOFS accomplishes this by continuously assessing feature utility against the most recent data window, dynamically elevating newly pertinent features and demoting or discarding those whose statistical relevance or correlation with the target concept has diminished.

Thirdly, anomaly or intrusion detection systems often face limited labelled data and few attack samples; AOFS may operate in both supervised and unsupervised settings to identify attributes that effectively distinguish abnormal or zero-day attack patterns [127]. AOFS mitigated overfitting by focusing the model on the most robust and generalizable features, thereby improving performance despite small training sets [128]. More critically, in fully unsupervised learning, common for detecting novel threats, AOFS is indispensable. It can employ criteria such as variance, feature entropy, or outlier-induced distribution shifts to identify and select features that provide the most significant separation between the bulk of the data and potential anomalies, thereby directly improving the sensitivity and clarity of anomaly scores without labelled examples.

Below, we survey the adoption of various strategies for feature/dimension processing across Online AutoML models. While all frameworks acknowledge the importance of effective feature handling in dynamic and high-dimensional cybersecurity environments, the level of methodological transparency varies substantially across studies.

DDoSNAS [129] adopted an ensemble feature selection approach combining Random Forest, Gradient Boosting, XGBoost, LightGBM, and CatBoost, followed by Recursive Feature Elimination (RFE) to iteratively rank and prune low-importance features. In contrast, Maddu et al. [119] relied on domain-adapted feature extraction by fine-tuning EfficientNet-B0 on IIoT traffic, enabling automated learning of hierarchical, domain-specific representations.

The authors of [114] modeled network flows as multidimensional stochastic processes using a variational autoencoder (VAE), compressing inputs into probabilistic latent variables parameterized by mean and variance. Ref. [61] employs an autoencoder with a Cluster-Repelling Contrastive (CRC) loss to enhance representation separability under limited or incremental data. Meanwhile, Maddu et al. [119] further leveraged SimCLR-based self-supervised learning to generate robust embeddings in low-label regimes.

Additionally, DDoSNAS [129] introduced separate embedding layers and Transformer encoders for macro- and micro-level decision representations, thereby enabling hierarchical reasoning during architecture search. Temporal dependencies in traffic patterns are captured by Anupama Babu et al. [116] using bidirectional GRUs with attention mechanisms.

Input features typically include flow-level and statistical traffic attributes, such as packet and byte counts, average packet size, protocol information, entropy, and inter-arrival times [114]. From a computational perspective, frameworks range from lightweight designs (e.g., DDoSNAS with ∼94k FLOPs and a 276 KB model) to enterprise-scale adaptive systems that require multi-GPU infrastructure [62], though most report inference latencies below 15 ms, making them suitable for near-real-time deployment.

Despite these advances, many studies provide limited details on preprocessing steps, feature dimensionality, categorical encoding, and feature importance computation, constraining reproducibility and cross-framework comparison.

Flow-level and statistical traffic characteristics, such as packet and byte counts, average packet size, protocol information, entropy, and inter-arrival times, are examples of input features [114]. From a computational standpoint, frameworks range from extremely lightweight designs (e.g., DDoSNAS with ∼94k FLOPs and a 276 KB model) to enterprise-scale adaptive systems requiring multi-GPU infrastructure [62]. However, most reports indicate inference latencies below 15 ms, making them suitable for deployment in near-real time.

Reproducibility and cross-framework comparisons are hindered by the fact that many studies provide scant information on preprocessing procedures, feature dimensionality, categorical encoding, and feature importance computation.

### 5.4. Online Model Selection and Training

In streaming environments, data arrive as high-velocity streams, demanding models that are constantly updated [130], and this concept applies to IoT and EV charging network systems. Here, OMS often entails maintaining a collection of potential models, ready to adjust/evolve as new data arrive [130]. Popular methods use incremental learners, such as Hoeffding Tree classifiers, either individually or in groups. In addition to online incremental learners, Zhang et al. [117] proposed an adaptive dimension that uses an autoencoder and latent input features. Additionally, in Adaptive Random Forests (ARF), several Hoeffding Trees are trained on randomized feature subsets, each with its own drift detector. When a tree’s performance, as determined by a technique such as ADWIN, declines, it is replaced with a newly initialized tree trained on the current data. In this approach, the pool of classifiers is managed dynamically, with underperforming or failing models retired regularly and new candidate models added and used to keep the ensemble up-to-date [130].

Methods such as ARF and bagging help maintain an online decision tree forest. Every tree is refreshed instance-by-instance, with drift monitoring; if the error level increases, it is replaced/reset [131]. Hoeffding Adaptive Trees (HAT) extend this concept by incorporating drift checks directly at leaf nodes. Generally, the collection of models is kept varied (e.g., by using random feature subspaces or various tree variations), and weak performers are renewed or pruned to account for shifting distributions [130].

Streaming options are available for classical ensemble architectures. Online bagging and boosting use Poisson weighting to imitate bootstrap sampling for every new case. In the context of online boosting, the base learner is incrementally updated with instance weights, which improve for previously misclassified cases [132]. These approaches intrinsically monitor model performance: misclassification-triggered weight updates, thereby concentrating new learners on “hard” cases. Like ARF, online boosting approaches analyze learners’ mistakes regularly and may remove or replace poor models if their contributions fall below a certain threshold.

Streaming deep learning models, such as small neural nets or Deep Neural Networks (DNNs) trained incrementally, are an emerging field. In reality, deep nets need to be trained with extreme caution to prevent catastrophic forgetting [133,134,135]. Deep models are being adapted online using one-pass (incremental) training, regularization, replay buffers [133,135], and dynamically expandable architectures. Deep approaches, on the other hand, often demand more compute and memory, making them less frequent in lightweight IoT settings. Adapting constant-learning methods to streaming data [135] is a promising yet demanding area of study.

### 5.5. Hyperparameter Optimization in Streaming Context

HPO faces unique challenges that extend well beyond those in static contexts [136,137] when applied to streaming and non-stationary environments [138,139]. In Online AutoML, hyperparameters must be tuned not only for efficiency but also for latency, memory, and computation while remaining responsive to changes in the underlying data and concept drift. These evolving conditions render traditional offline HPO methods inadequate, thereby motivating the development of adaptive strategies, including dynamic CASH, hyperparameter reuse, and lightweight, real-time optimization mechanisms. While these methods are technically demanding, they are also crucial for ensuring that automated machine learning remains robust and applicable in real-world, continuously changing scenarios.

Traditionally, the CASH problem is formulated using a static dataset, in which the data distribution is assumed to remain fixed [66,88]. The goal is to identify the optimal learning algorithm and its hyperparameters to minimize a specified loss function. However, this classical approach falls short in streaming environments, where data arrive continuously, and patterns can shift over time. As a result, the optimization process must become more flexible and responsive to these ongoing changes.

Online CASH extends the classical CASH formulation to non-stationary data streams by explicitly incorporating time, resource constraints, and adaptation dynamics. In practical terms, Online CASH is guided by three main priorities:

1. Efficiency—preferring fast, approximate, or incremental methods over exhaustive searches;
2. Stability—aiming to avoid frequent, unnecessary re-tuning when only minor or temporary changes occur.
3. Responsiveness—ensuring that the system can quickly adapt when truly significant changes or drifts are detected.

As evidenced by current Online AutoML systems, Online CASH is often realized implicitly through reinforcement learning-based parameter control, evolutionary multi-objective optimization, or gradient-driven online updates, rather than by explicitly re-solving the full CASH problem at each time step. Nonetheless, the formalization of Online CASH provides a unifying framework for analyzing and comparing adaptive AutoML strategies in streaming machine learning.

#### 5.5.1. Online CASH Formulations and Architectural Optimization

Only a limited subset of surveyed models explicitly formulates the learning problem as an online extension of the CASH principle. The DDoSNAS framework [129] is the most comprehensive approach, framing the problem as the joint optimization of neural architecture and hyperparameters under real-time constraints. It employs a Transformer-controlled Neural Architecture Search (NAS) mechanism that simultaneously optimizes accuracy, latency, and FLOPs through Pareto-based multi-objective search. Efficiency is achieved through one-shot weight sharing, enabling the evaluation of architectural alternatives without repeated retraining. In contrast, most other frameworks assume fixed model architectures and focus exclusively on parameter-level adaptation, thereby addressing only a partial subset of the CASH formulation.

The IDNet framework [116] is another notable example that integrates Katib-based hyperparameter optimization into an MLOps pipeline. While it does not dynamically optimize model architecture, it supports automated hyperparameter tuning and redeployment, thereby illustrating a pragmatic yet narrower interpretation of online CASH.

#### 5.5.2. Hyperparameter Reuse and Adaptive Optimization

Given the computational cost of repeated hyperparameter search in streaming environments, several models adopt hyperparameter reuse and incremental optimization strategies. DDoSNAS implicitly reuses hyperparameters through its weight-sharing mechanism, avoiding retraining from scratch when evaluating candidate architectures. Adaptive frameworks, such as that of Thangatharani et al. [62,129], rely on reinforcement learning to continuously adjust detection parameters, thereby enabling fine-grained adaptation without explicit search restarts.

In IDNet, hyperparameter reuse is governed by a sliding-window strategy, where previously optimized configurations remain active until invalidated by drift signals. This event-driven optimization paradigm ensures that expensive retraining is triggered only when statistically justified, rather than on fixed schedules [116].

#### 5.5.3. Drift-Aware Optimization and Triggering Mechanisms

Drift awareness plays a critical role in determining when hyperparameter configurations become obsolete. Explicit drift detection is relatively rare; IDNet stands out by employing the Population Stability Index (PSI) to detect distributional changes and trigger retraining and re-optimization [116]. Most other frameworks rely on implicit drift handling, in which performance degradation or evolving data characteristics indirectly drive parameter adaptation.

For instance, reinforcement learning-based adaptive frameworks treat changing network behaviours as part of the environment dynamics, allowing hyperparameters to evolve continuously without discrete drift alarms [62]. Similarly, the VAE–Kalman system proposed by Vladov et al. [114] performs continuous parameter updates using an Euler gradient rule, effectively embedding drift adaptation within the learning process rather than separating detection and response.

#### 5.5.4. Efficiency Constraints and Practical Deployment

Across all models, computational efficiency is a dominant design constraint. DDoSNAS explicitly optimizes for ultra-low resource usage, achieving sub-millisecond latency and minimal FLOPs, making it suitable for on-device deployment [129]. Other frameworks report real-time processing latencies ranging from 1.5 ms to under 100 ms, but often lack detailed analysis of the overhead introduced by hyperparameter optimization itself.

Taken together, these components show that Online AutoML depends on coordinated adaptation across the full pipeline, motivating a review of existing frameworks, libraries and their limitations in real-world IoT and EV charging systems.

### 5.6. Online AutoML in IoT and EV Charging Network Security

The datasets currently used in EV-charging cybersecurity research provide a strong foundation for applying Online AutoML in real-world intrusion-detection pipelines. In particular, datasets such as CICEVSE2024, the OCPP-centric hybrid dataset, and the PowerBench EVCS datasets contain multi-modal, continuously evolving data streams that naturally align with streaming and adaptive learning approaches [140,141,142,143].

First, online preprocessing is essential due to the heterogeneous nature of EV-charging data. For example, datasets such as CICEVSE2024 include power-consumption values, network traffic (PCAP/CSV), and host-level activity logs collected during both idle and charging states [140,141]. These data streams often exhibit noise, missing values, and temporal inconsistency, particularly when charging behaviour changes dynamically. Online normalization, incremental feature extraction, and streaming noise filtering, therefore, become necessary components of an Online AutoML pipeline.

Second, drift detection is particularly important in EV and IoT environments. The datasets described in the literature explicitly include multiple operating states (e.g., idle versus charging), evolving protocol behaviour (e.g., OCPP 1.6 to OCPP 2.0), and diverse attack scenarios such as reconnaissance, denial-of-service (DoS), and backdoor attacks [140,142]. These variations indicate both concept drift (changing traffic and attack patterns) and data drift (changes in feature distributions). Online drift-detection mechanisms can therefore be used to trigger adaptive retraining whenever charging behaviour, firmware behaviour, or protocol usage evolves.

Third, online feature selection is necessary because modern EV-charging datasets are increasingly high-dimensional. For instance, the OCPP-centric hybrid dataset contains more than 55 protocol-level and timing-based features, including state-machine transitions and payload-level characteristics [142]. Processing all features continuously in a resource-constrained IoT environment is inefficient; therefore, streaming feature-selection methods can dynamically retain only the most informative network, behavioural, and power-related features for intrusion detection.

Fourth, online model selection and training enable continuously adapting IDSs. Since the datasets contain both real testbed data (e.g., CICEVSE2024) and simulated power-system attack scenarios (e.g., PowerBench EVCS), the statistical properties of the data differ significantly across environments [140,143]. Online AutoML can therefore automatically switch among models, such as incremental tree ensembles, anomaly detectors, and adaptive classifiers, based on real-time performance.

Finally, streaming hyper-parameter optimization (HPO) is essential for balancing detection accuracy with latency and resource constraints. EV charging stations and IoT devices typically operate under limited computational resources, yet the datasets demonstrate large-scale traffic, multi-modal features, and multiple attack types [140,142,143]. Streaming HPO enables Online AutoML systems to dynamically adjust model complexity, learning rates, and drift-detection sensitivity to maintain high detection performance while respecting real-time operational constraints.

Overall, the structure and characteristics of current EV-charging cybersecurity datasets strongly support the use of Online AutoML, particularly for adaptive intrusion-detection systems capable of operating under continuously evolving network and charging environments [140,141,142,143].

## 6. AutoML and Online Learning Frameworks in Research and Industry

The rapid expansion of data-intensive applications has necessitated the development of frameworks that automate machine learning workflows and adapt to dynamic data environments. AutoML and online/incremental/stream learning frameworks address these requirements, providing tools for model selection, hyperparameter optimization, and real-time adaptation. These frameworks, listed in subsections below, are increasingly adopted across research and industry, enabling scalable, efficient, and user-friendly machine learning solutions.

### 6.1. Leading AutoML Frameworks

AutoML frameworks simplify and automate the traditionally labour-intensive process of developing ML models. Key frameworks include:

- Auto-sklearn: Python-based, integrates with scikit-learn, automates model selection and hyperparameter tuning [26,144].
- Tree-based Pipeline Optimization Tool (TPOT): Uses genetic programming to optimize pipelines, compatible with Python/scikit-learn [26,145].
- H2O AutoML: Scalable and open-source, supports classification/regression, widely used in business analytics [83,145].
- AutoGluon: A freely available AutoML framework built by Amazon that streamlines the development of ML models with minimum user input [146,147].
- AutoKeras: Deep learning AutoML, built on Keras/TensorFlow, user-friendly for non-experts [148].
- Google Cloud AutoML: Cloud-based AutoML for tabular, image, and text data, minimal coding required [84].
- Auto-WEKA: AutoML for the WEKA platform, focusing on model and hyperparameter selection [149].

### 6.2. Online Learning Frameworks

For real-time applications and continuously evolving datasets, online or incremental learning frameworks provide essential capabilities:

- River: Python library for streaming data and continual learning [150,151].
- Massive Online Analysis (MOA): Java-based, efficient for large-scale stream learning [152,153].
- Scikit-Multiflow: Python implementation of MOA algorithms, now largely replaced by River [151,153].
- Stream-learn: Handles concept drift, imbalanced data, and batch/online evaluation [154].
- Kafka-ML: Manages ML pipelines over data streams, supports online/distributed learning [155,156].
- CapyMOA: Integrates MOA and PyTorch, hybrid online/deep learning [153].

Table 3 demonstrates how AutoML platforms like Auto-sklearn, H2O, TPOT, and AutoGluon emphasize user-friendliness via advanced abstractions, which include Python APIs, single-line training commands, and graphical UIs, such as H2O Flow or cloud-based dashboards like Google Cloud AutoML [157,158]. These solutions reduce the demand for extensive programming knowledge by automating workflows (model selection and hyperparameter optimization), thereby increasing accessibility for both newcomers and corporate users. Conversely, online learning frameworks such as River, MOA, and Scikit-Multiflow are primarily developer-centric, emphasizing programmatic handling, streaming data management, and incremental updates. Although they require more technical expertise, they enable adaptability to real-time and dynamic learning environments. Also, streaming frameworks such as River and Kafka-ML provide real-time monitoring via REST APIs and web UIs [156,159].

From an efficiency standpoint, AutoML platforms prioritize robust automation and predictive performance, which often leads to increased computational overhead [144,145]. AutoML technologies such as AutoGluon achieve superior accuracy and efficiency through optimized pipelines. In contrast, alternatives like TPOT and AutoKeras may exhibit slower performance or higher resource consumption due to evolutionary search or DL optimization. H2O AutoML and cloud-based systems can scale efficiently but need significant infrastructure. Conversely, online learning frameworks like River and Kafka-ML are optimized for efficiency in streaming contexts, providing low-latency updates, immediate processing, and enhanced adaptability for resource-limited or perpetually changing data environments, especially where dynamic management of concept drift is essential [117,150,166].

It is noteworthy that, although many libraries exist for AutoML and online learning, there are no libraries for Online AutoML. To a large extent, the situation underscores the strong research potential of integrating AutoML and online learning libraries into a comprehensive Online AutoML framework. Indeed, this is a promising research direction.

## 7. Adversarial Attacks and Defences on Online AutoML

AML has matured into a well-established and rapidly expanding research field, supported by extensive studies on both attack strategies and defensive mechanisms. Malik et al. [58] present a comprehensive systematic review that highlights substantial progress across multiple application domains, with early experimental work predominantly focused on computer vision. Malik et al. [58] introduces a detailed taxonomy of AML attacks, defences, and enabling technologies, framing adversarial risk as a life-cycle–wide concern rather than a purely model-centric vulnerability. In this taxonomy, attacks are classified into evasion, poisoning, and privacy-based categories, while corresponding defences are mapped to different stages of the secure AI development life cycle. The primary contribution of this survey is to synthesize fragmented AML research into a unified AI assurance perspective suitable for real-world organizational deployment.

More recently, the focus of AML research has shifted away from image-based benchmarks toward security-critical domains, particularly cybersecurity. Increasing attention is being paid to network security, IDSs, federated learning, and IoT environments. Contemporary studies investigate adversarial behaviour in operational settings, including network traffic analysis, PDF malware detection, smart grids, O-RAN cellular networks, and adaptive control or recommender systems [56,167]. In particular, Ghosh et al. [56] examine AML threats targeting network security systems, demonstrating how evasion, poisoning, and model inversion attacks compromise IDSs and traffic classification pipelines. Their analysis provides domain-specific insights into adversarial tactics against deployed network defences. It exposes the limitations of widely adopted countermeasures—including adversarial training, defensive distillation, and randomized smoothing—when faced with adaptive attackers. Collectively, these findings underscore that threat models affecting security-critical, online systems differ fundamentally from those assumed in static vision datasets.

Recent real-world incidents further illustrate the practical impact of AML attacks on deployed security systems. The Proofpoint EchoSpoofing campaign, reported between January and June 2024, successfully bypassed machine-learning-based email security filters, resulting in large-scale credential theft and financial fraud [168]. Additionally, reports from late 2025 document AI-assisted cyberattacks in which large language models were used to automate reconnaissance and exploitation tasks, signalling a marked escalation in both the sophistication and automation of adversarial capabilities [169].

Controlled experimental studies corroborate these real-world observations by demonstrating significant performance degradation of IDS models under adversarial conditions. In the context of in-vehicle intrusion detection, multiple studies report accuracy drops ranging from 18 to 60 percentage points, with state-of-the-art models declining from approximately 97% to 79% in F1 score under black-box attacks [170,171]. Although various defence mechanisms have been proposed, their effectiveness remains limited. Adversarial training often reduces attack success rates by only around 30%, and evaluations of commercial IDS solutions reveal that a substantial fraction of adversarial evasion attempts continue to evade detection [171,172].

These findings raise critical concerns for modern IDS deployments, particularly those based on Online AutoML and continuous learning. Unlike static models, Online AutoML systems continuously adapt their feature representations, model architectures, and hyperparameters in response to streaming data. While such adaptability is essential for coping with evolving traffic patterns, it also expands the attack surface by enabling adversaries to influence model updates over time. In this setting, adversarial inputs may not only evade detection at a single time step but also progressively bias the learning process, leading to sustained degradation of detection performance.

Consequently, securing Online AutoML-based IDSs requires a shift in perspective beyond protecting individual classifiers. The entire adaptive pipeline, including drift detection, model selection, and hyperparameter optimization, must be considered as part of the threat model. This is particularly important in Online AutoML, where attacks may affect not only a deployed ML model, but also the adaptive logic that governs feature updates, model replacement, and hyperparameter reconfiguration over time. Understanding which AML attack types can exploit these adaptation mechanisms and how such attacks affect detection accuracy over time is essential for designing robust IDS frameworks for streaming and IoT environments.

### 7.1. Adversarial Attacks on Online AutoML

The fundamental theory of AML rests on the observation that contemporary ML models, particularly DNNs, are intrinsically susceptible to adversarial instances; inputs that have been subtly and deliberately altered to induce misclassification while remaining nearly indistinguishable from genuine data to the human eye. Goodfellow et al. [173] provided a crucial theoretical elucidation of this issue, ascribing the vulnerability of neural networks not to overfitting or nonlinearity, as previously conjectured, but to their significant linearity in high-dimensional spaces. Introduced by Goodfellow et al. [173], the Fast Gradient Sign Method (FGSM) is a computationally efficient method for creating adversarial instances by perturbing data (input) towards the gradient of the loss function’s direction relative to the input, hence maximizing the prediction accuracy of the model error.

Ghosh et al. [56] situated AML within network security systems, emphasizing the tangible threats posed by adversarial attacks, including evasion and poisoning, that may jeopardize essential applications, such as medical diagnostics and autonomous vehicles. They underscored the imperative for strong deployment standards and present a classification of AML attacks, comprising evasion, poisoning, and privacy-focused attacks—each characterized by distinct strategies and ramifications for system integrity, confidentiality, and availability. Additionally, Malik et al. [58] provided an extensive overview and classification of AML attacks and countermeasures, differentiating between black-box and white-box attack strategies and detailing techniques such as FGSM, Projected Gradient Descent (PGD), and Carlini–Wagner (CW) attacks. They assessed a range of defensive measures—adversarial training, formal verification, randomized smoothing, and input reconstruction—while acknowledging ongoing difficulties with generalizability, scalability, and the lack of uniform evaluation metrics.

Many existing surveys [56,58,173] broadly classify AML attacks into three main categories: evasion, poisoning, and privacy-based attacks. These works provide the foundational theoretical background. Instead, the following sections focus on specific attack types commonly employed against IDSs, with particular emphasis on their relevance and implications for Online AutoML-based models. Based on the above attack types, we present studies and evaluations conducted in the last 5 years on Online AutoML models across diverse fields.

- Evasion Attacks: Evasion attacks manipulate inference-time inputs to induce misclassification without altering model parameters. They are commonly categorized into white-box, black-box, and transfer-based variants. In IDS contexts, evasion typically involves crafting network traffic, malware artifacts, or control signals that remain functionally valid while bypassing detection mechanisms [57]. In Online AutoML–driven IDSs, particularly within IoT systems, evasion attacks are especially problematic because adaptive learning mechanisms may misinterpret adversarial inputs as benign concept drift, thereby reinforcing attacker-induced patterns over time.
  Several studies examine evasion in real-time or dynamic systems. Gong et al. [59] demonstrate that adversarial examples can be generated under strict latency constraints, invalidating assumptions that real-time requirements inherently protect deployed systems. Joe et al. [174] extend evasion into the temporal domain, showing that recurrent models can be compromised by “hallucinating the future”, resulting in long-term degradation through carefully crafted input sequences. In networked environments, Fladby et al. [57] show that ML-based IDSs can be evaded via minimal feature perturbations. In contrast, Apruzzese et al. [175] demonstrate that evasion effectiveness increases significantly when combined with concept drift. Amoeba [176] employs reinforcement learning to generate adversarial network flows that achieve high evasion success rates without prior knowledge of the target model, posing a direct threat to black-box IDS deployments. Additional application-specific evasion studies include EvadeRL for PDF malware detection [177] and reinforcement learning-based attacks on censorship, recommendation and control systems [178,179]. Collectively, these works illustrate that adaptive evasion strategies generalize across domains and are particularly effective against continuously learning systems.
- Poisoning Attacks: Poisoning attacks target the training or update process by injecting malicious samples, labels, or gradients. Common forms include availability poisoning, targeted poisoning, backdoor insertion, and model poisoning. In online and federated learning settings typical of IoT deployments, such attacks can be introduced gradually, making them difficult to detect [180]. Online AutoML pipelines further amplify this risk by continuously adapting model architectures and hyperparameters with limited validation, enabling poisoned data to distort decision boundaries persistently. Poisoning attacks are extensively studied in federated and online learning contexts. Chen et al. [180] analyze backdoor poisoning in federated meta-learning and show that malicious behaviours persist even after additional training rounds. Panigrahi et al. [181] introduce double-momentum backdoor attacks that exploit optimizer dynamics to maintain high backdoor accuracy while preserving benign task performance. Wen et al. [182] and Zhang and Huang [183] demonstrate that Byzantine-robust aggregation rules are insufficient against adaptive poisoning strategies. Wang and Zuccon [184] analyze poisoning in federated online learning-to-rank systems. At the same time, Zhu et al. [185] reveal the susceptibility of edge AI in IoT-enabled smart cities to online data poisoning.
- hlPrivacy-Based Attacks: Privacy-based attacks aim to extract sensitive information encoded within trained models rather than directly impairing prediction accuracy. While less immediately disruptive to IDS performance, these attacks pose significant risks in IoT environments, where models may implicitly capture sensitive behavioural, infrastructural, or user-specific information [186]. System-level analyses further expose privacy and architectural vulnerabilities. Feng et al. [186] show that stateful defences remain susceptible to black-box attacks, while Chiejina et al. [167] evaluate AML threats and defences in O-RAN-based cellular networks. These studies highlight that model state, memory, and adaptation logic can themselves serve as attack surfaces—an especially critical issue for Online AutoML systems.

### 7.2. Adversarial Defences and Online AutoML

The range of adversarial threats is extensive, including evasion, backdoors, privacy violations, data and model poisoning, and others. Consequently, a variety of defensive strategies have been proposed, each characterized by distinct principles, advantages, and shortcomings [187]. This section offers a thorough examination of prevalent AML defensive methods, concentrating on the following categories. Table 4 contains the advantages and disadvantages of the discussed defence strategies.

- Adversarial Training (AT): AT is a prevalent and experimentally effective method for defending against adversarial instances. The technique entails enhancing training with adversarially modified inputs, thereby compelling the model to establish robust decision boundaries [188]. The procedure is generally formulated as a min-max optimization problem, in which the inner maximization yields adversarial cases. At the same time, the outer minimization adjusts the ML model parameters to reduce the loss on these instances.
- Detection/Input Transformation (DIT) [189,190,191]: The goal of this defence type is to detect or eliminate adversarial instances before reaching the model. Some of the techniques used include feature squeezing [189] and detection using out-of-distribution [190] or autoencoder (MagNet) [191] approaches. During feature squeezing, the adversary’s search space is constrained to identify differences between the initial and altered predictions by reducing input complexity (e.g., spatial smoothing, colour depth) under detection. For detection using statistical methods, the aim is to flag inputs that deviate from the distribution of clean data using Gaussian discriminant analysis, the Mahalanobis distance, principal component analysis, or other statistical metrics. In autoencoder-based detection, the goal is to identify adversarial cases by reconstructing the inputs and calculating the reconstruction error (MagNet).
- Discrete Adversarial Optimization (DAO): DAO broadens AT to encompass domains characterized by discrete input spaces such as categorical and text data, in which gradient-based attacks exhibit less efficacy [192]. This methodology redefines adversarial sample generation as a discrete optimization problem, often employing reinforcement learning or combinatorial search techniques. This defence strategy improves robustness but incurs substantial computational overhead, often incompatible with real-time IoT constraints [192].
- Federated Defences: Federated Learning (FL) offers a decentralized structure in which several clients jointly train a common model without the transfer of raw data. Although FL improves privacy, it also introduces new vulnerabilities, including poisoning, Sybil, backdoor, and inference attacks. Therefore, Federated defences (FD) are specific methods for safeguarding FL platforms against these cybersecurity risks, often by integrating strong anomaly-detection, aggregation, privacy-preserving, and secure-hardware strategies. Federated defences, including secure aggregation and attestation, reduce poisoning impact but fail to mitigate adaptive adversaries fully [193].
- Gradient Obfuscation and Masking (GOM): These defensive measures aim to impede attackers’ ability to compute gradients for the purpose of executing attacks [194]. This strategy is effective against gradient-based attacks, such as Projected Gradient Descent (PGD) attacks [195]. Although GMO strategies may temporarily reduce attack success rates, they often provide only surface-level resilience and are susceptible to other adaptive attacks. In other words, other vulnerabilities might still exist even after the application of this defence measure. Manifestations of GOM include shattered and stochastic gradients as well as non-differentiable operations [194].
- Hardware-assisted Defences: Hardware-assisted defences utilize hardware capabilities or trusted execution environments (TEEs) to offer an extra layer of security against adversarial attacks. These defences operate at the intersection of ML and hardware security, seeking to identify, avert, or mitigate threats by observing low-level system activity or by segregating critical calculations. For instance, Pelta [196] leverages trusted execution environments to isolate models, though deployment cost and scalability remain barriers.
- Moving Target Defences (MTDs): MTDs are a category of AML defence strategies that dynamically modify the attack surface within an ML application, hence complicating adversaries’ efforts to devise effective attacks [197,198]. In contrast to static defences, MTDs provide uncertainty and variety by regularly altering parameters, inference paths, and architectures, thus diminishing the transferability and repetition of adversarial cases. MTDs, such as Morphence [197], introduce model diversity to reduce attack success rates but significantly increase system complexity.
- Input Sanitization and Data Provenance (ISDP): These measures seek to identify and eliminate contaminated or irregular data from datasets required for model training, thereby countering model and data poisoning assaults. These approaches function by detecting anomalies or dubious data patterns before model training [199]. Regarding data provenance, the aim is to preserve lineage information for every data point, facilitating the detection of dubious or altered samples [200].
- Ensemble and Model Randomization Defences (EMR): These defences use several distinct models or randomized inference paths to diminish the transferability and efficacy of adversarial cases. An example of this technique is the Negative Correlation Ensemble (NCEn) [201], randomized model selection, and input and training diversity. By enhancing variety and unpredictability, these defence strategies complicate attackers’ ability to develop generally efficient perturbations.

At a higher level, frameworks such as MITRE ATLAS, the NIST AI Risk Management Framework and toolkits including IBM ART, Microsoft Counterfit, and the IBM AI Privacy Toolkit promote a shift-left security philosophy by embedding AML considerations throughout the AI life cycle. However, these frameworks remain largely advisory and do not directly resolve the technical challenges posed by autonomous, continuously adapting AutoML systems. Table 4 contains AML defences, their techniques, advantages, and disadvantages.

**Table 4:** Summary of AML defence types, techniques, advantages, and disadvantages.

AML Defences	Techniques	Advantages	Disadvantages
Adversarial Training [188]	Adversarial instances, min-max optimization	Robust empirical performance	Accuracy trade-off, substantial training costs.
Detection/Input Transformation [189,190,191]	Feature squeezing, statistical (OOD)/autoencoder-based detection	Easy integration, model-agnostic	Susceptible to adaptive cyberattacks, seemingly accuracy-degrading.
Discrete Adversarial Optimization [192]	RL, search (combinatorial, best-first)	Bypasses gradient dependence, efficient in specific cases	High computational costs, task-based optimization.
Ensemble and Model Randomization Defences [201]	Negative correlation, involves several models, diversity of input	Enhances robustness, minimizes transferability	Substantial resource consumption, complexities in deployment.
Federated Defences [193]	TEEs, Robust aggregation, anomaly detection	Preserves privacy, scalable, guards against multiple attacks	Maybe hardware dependent, domain-specific, computational overhead.
Gradient Masking/Obfuscation [194,195]	Non-differentiable layers, randomization	Initial deterrence, simple	False sense of security, fails under adaptive attacks.
Hardware-assisted Defence (Pelta) [196,202]	TEEs, hardware counters, gradient concealing	Low computational overhead, hardware-oriented safeguards	Hardware-dependent, memory limitations.
Input Sanitization/Data Provenance [199,200]	Provenance monitoring, anomaly detection	Eliminates contaminated data, strengthens trust	Susceptible to orchestrated cyber-attacks, may discard legitimate data.
Moving Target Defences (MTDs, Morpheus) [197,198]	Randomized inference, Morphing	Heightens adversary uncertainty	May involve high computational overhead and require tuning.

### 7.3. Online AutoML Maturity Across AML Studies

A key consideration when screening and reviewing AML studies in the context of Online AutoML is the extent to which the evaluated models are both online and automated in their learning and adaptation processes. This paper examines the extent to which the systems studied can be classified as fully Online AutoML. To systematically assess this distinction, the reviewed studies are categorized along three orthogonal dimensions:

1. Online vs. Offline Learning (sequential or streaming updates versus static training),
2. Automated Adaptation Components (automated model, hyperparameter, or pipeline optimization), and
3. Drift Detection and Adaptation (explicit mechanisms for detecting and responding to concept or data drift).

Table 5 summarizes the Online AutoML maturity of various adversarial ML studies across three dimensions: online learning, automated adaptation, and drift detection/adaptation. This categorization exposes a disconnect between adversarial ML research and the requirements of fully autonomous Online AutoML systems for IDS and IoT environments.

### 7.4. High Online AutoML Completeness

Few studies achieve high completeness by including all three key aspects.

Chen et al. [180] propose a federated meta-learning framework with iterative updates that, while not operating on classical high-frequency streams, enables automated adaptation through meta-learning (Reptile) and local fine-tuning. Adaptation is triggered continuously based on performance degradation, implicitly addressing drift in federated environments. Mao et al. [177] introduce EvadeRL, a system that supports online fine-tuning with sequential data and automated model adaptation. Over time, it adapts to changes in malware behaviours and detector responses. Moskalenko et al. [203] propose an online framework that uses self-knowledge distillation, hierarchical labelling, and consistency regularization. Their method includes concept-drift detection and event-driven adaptation over more than 200 iterations, making it one of the few studies at the time to demonstrate drift-aware automated adaptation in practice. Together, these three studies are among the clearest examples of high Online AutoML completeness used in AML studies, to the best of our knowledge, as they combine online learning, automated adaptation, and drift-aware methods.

### 7.5. Moderate Online AutoML Completeness

Many other studies support online learning and some automation, but they do not include explicit or robust drift detection. Amich and Eshete [197] propose Morphence, a moving-target defence that processes sequential queries and automates model-pool renewal based on prediction confidence. However, adaptation is driven by time-based scheduling rather than explicit drift detection. Feng et al. [186] introduce OARS-style hyperparameter adaptation in a sequential setting, enabling automated defence responses based on system feedback. While adaptation is continuous and event-driven, drift is not explicitly modelled. Azizi Ariffin et al. [193] incorporate online edge updates with adaptive aggregation to mitigate adversarial attacks. Although the system detects adversarial behaviour and adapts accordingly, it does not implement general AutoML mechanisms beyond security-focused aggregation. Ivgi and Berant [192] operate in a fully online setting with continuous adversarial augmentation at every training step. However, no automated adaptation logic or explicit drift detection is incorporated. These studies demonstrate that online behaviour alone is insufficient to claim strong Online AutoML completeness when automated adaptation and drift-aware control remain limited.

### 7.6. Limited Online AutoML Completeness

Some studies use online learning but do not include AutoML features or clear drift modelling. Zhang and Huang [183] considers lifelong and imitation learning with sequential updates as online learning but do not introduce automated adaptation mechanisms. Drift handling remains implicit and periodic. Panigrahi et al. [181] analyze poisoning across sequential federated learning rounds but do not incorporate any AutoML or drift-aware adaptation components. Gong et al. [59] demonstrate adversarial attacks under real-time streaming constraints. As low as 0.01 s, yet adaptation remains manual and drift handling is implicit. Liu et al. [176], Wang and Zuccon [184], and Joe et al. [174] all operate in real-time or continuously updated environments, but none introduce automated model selection, hyperparameter optimization, or explicit drift detection.

### 7.7. Minimal Online AutoML Completeness

Finally, several system-level studies lack online learning or AutoML capabilities altogether. Chiejina et al. [167] analyze adversarial threats in O-RAN networks but do not explicitly implement online learning or automated adaptation, relying instead on interference-triggered responses. Queyrut et al. [196] focus on hardware-assisted isolation via trusted execution environments, without addressing online learning or drift adaptation. Apruzzese et al. [175] investigate the interaction between evasion attacks and concept drift but stop short of implementing online learning or adaptive mitigation mechanisms.

Across the studies reviewed, only three studies, Chen et al. [180], Mao et al. [177], and Moskalenko et al. [203], show high Online AutoML completeness by combining online learning, automated adaptation, and drift-aware methods. About 65% of studies use online learning but lack full AutoML features, and clear drift detection and adaptation are uncommon. This shows a gap between current adversarial ML research in fully autonomous Online AutoML systems. In IDS and IoT settings, this gap increases AML risk, underscoring the need for Online AutoML designs that handle adversarial behaviour, concept drift, and automated optimization together in a more unified and robust manner.

### 7.8. Adversarial Attacks and Defences in IoT & EV Charging Networks

In this section, we discuss examples of how attackers can leverage adversarial attacks and defence mechanisms to compromise Online AutoML models deployed within IoT and EV charging network environments. Given the understanding and intricacies discussed in the previous sections, this scenario is highly plausible and warrants further examination here. These attacks [57,59,167,174,176,177,178,180,181,204] take on additional significance and distinctive structural features due to the implementation of Online AutoML in security-sensitive areas of contemporary linked infrastructures, such as IoT and EV networks. To cope with their real-time operational activities, one must consider their similarities; both systems increasingly use online, federated, or adaptive ML pipelines [8,16]. They also share a basic dependence on constant data streams from remote sensors and other endpoints.

The relationship between these attack types and the deployment scenarios of both the IoT and EVCS reveals a set of structural deficiencies. First, the feedback generated by streaming AutoML can serve as a conduit for contamination. IoT devices and EV charging stations generate ongoing implicit input (e.g., meter readings, traffic categorizations, anomaly descriptions, and click indicators) that Online AutoML systems use to retrain. Any attack(es) that utilize this kind of feedback stream—ranging from the manipulation of concept drift [175] down to the poisoning attacks of reverse simulation [184] are consequently relevant in both networks. Secondly, aggregation in the FL manner can be a strategy for attack propagation. IoT and EVCS domains employ FL architectures to enable scalability and privacy, leading to the following: backdoor attack persistence in “federated meta-learning” [180], injection attacks that exploit momentum [181], and evasion attacks targeting “Byzantine-robust” defences [182]. Moreover, a backdoor introduced at a single IoT gateway, or at any EVCS, propagates to all connected devices that subsequently initialize from the compromised meta-learner. Thirdly, there may be physical ramifications of ML-driven logical attacks. In contrast to the majority of AI (machine learning) security scenarios, failures in IoT and EVCS have tangible real-world repercussions. A corrupted/poisoned IoT-driven ICS can damage equipment; an exploited anomaly detector (for EVCS) can facilitate grid-destabilizing repetitive load incidents. The RL-based evasion schemes [176,177,178] are not merely attacks that degrade accuracy in these circumstances; they also represent a critical step towards adversaries compromising physical infrastructure. Finally, resource limitations hinder the development of AI defence strategies. AML attacks on IoT platforms can result in device failure, service disruption, and the exploitation of personal data, making the development of safe and resilient AI models in the IoT domain a significant ongoing problem. IoT endpoints and EVCS edge processors generally lack the computational capacity to implement AT strategies, certified protections, or intricate query record monitoring. Specifically, the defences demonstrated to be most effective against attacks. Online AutoML, intended to operate under resource constraints, must identify robustness solutions that do not require the defensive architectures of stateful [186] or Byzantine-robust [182] defences.

Online AutoML provides a robust framework for implementing and enhancing AML defence strategies in dynamic streaming settings (IoT and EVCS), which exhibit traits such as decentralized sensing, live data streams, and diverse nodes. Firstly, AT methodologies [188] can greatly gain from Online AutoML by continually and automatically selecting resilient models in response to changing threat environments. In streaming IoT/EV contexts, attack patterns evolve; hence, Online AutoML may continuously retrain models on reweighted adversarial instances, ensuring that resilience adapts to new attack distributions rather than being fixed. Secondly, DAO [192] and recognized vulnerabilities like gradient masking [194] underscore the necessity for responsive model assessment. Online AutoML can alleviate these problems by consistently evaluating candidate models against adversarial verification and validation streams, thereby avoiding the selection of deceptively strong models that result from obfuscation. This corresponds to the necessity of circumventing deceptive defences [194]. Thirdly, for DIT protections, including feature squeezing [189], OOD identification [190], and MagNet [191], Online AutoML may autonomously choose and optimize AutoDP pipelines in response to streaming anomalies. In IoT/EV environments characterized by distributional drift, AutoML facilitates adaptable thresholding and feature modifications, thereby enhancing detection efficiency over extended periods without human recalibration. Fourthly, EMR defences [201] are intrinsically compatible with AutoML. Online AutoML systems may automatically create and enhance ensembles, thereby promoting variety and robustness while adapting to evolving attack techniques. This is particularly beneficial in electric-vehicle charging networks, as attackers may target specific models.

In federated contexts [193] relevant to dispersed IoT/EV systems, Online AutoML may improve safe aggregation and proactive learning by identifying optimal localized models and aggregation procedures in hostile environments. This enhances tolerance to poisoning while preserving scalability. HBDs such as Pelta [196] and attestation techniques [202] can be enhanced by AutoML, which optimizes models within secure/trusted zones to ensure model performance and integrity. Ultimately, ISDP procedures [199,200], together with MTDs [197,198], may be enhanced using Online AutoML to facilitate continual model/pipeline reconfiguration. This evolving adaptability is essential in IoT and EV technologies, where adversaries exploit static protection attributes. Considering these strategies, Online AutoML converts these defence strategies from fixed procedures into evolving, self-adjusting systems, markedly enhancing resilience in everyday cyber–physical situations. There is a need for rigorous experimentation to understand the technical and organizational impact of these Online AutoML-based defences.

## 8. Challenges and Research Directions

The reviewed literature indicates that current Online AutoML systems are promising but not yet sufficiently mature for robust deployment in dynamic IoT and EV charging environments, owing to several recurring challenges and limitations. These limitations span detection performance, computational efficiency, adaptability, evaluation methodology, and deployment scalability, collectively constraining the practical effectiveness of current approaches. Table 6 summarizes these challenges.

### 8.1. Performance and Accuracy Limitations

A prominent limitation of existing frameworks is their limited effectiveness in detecting previously unseen attacks. While many models achieve high accuracy, typically between 97% and 98%, on known attack classes, their performance declines substantially in zero-day scenarios. For instance, detection rates for novel attacks can drop to approximately 89.6% in some studies [62], highlighting a critical vulnerability in real-world deployments, where unknown threats are common.

A further challenge involves balancing task specialization and generalization. Highly optimized architectures, such as DDoSNAS, can achieve detection accuracies of up to 99.98%. Still, these models are tailored to specific attack categories, particularly DDoS, and do not support online adaptation [129]. This narrow focus limits their applicability in dynamic, heterogeneous threat environments.

False positive rates continue to pose a significant challenge. Some Online AutoML frameworks report false-alarm rates of 42.3%, which can overwhelm security analysts, increase operational costs, and diminish overall system effectiveness [62]. These results underscore the difficulty of achieving an optimal balance between attack sensitivity and practical precision in operational IDS deployments.

### 8.2. Computational and Resource Constraints

Online AutoML-based IDS frameworks frequently impose substantial computational requirements. Enterprise-scale adaptive systems may require up to 512 GB of RAM, eight Tesla V100 GPUs, and 128 CPU cores [62]. These resource demands significantly constrain deployment feasibility, especially for organizations operating in resource-limited environments.

Algorithmic bottlenecks also hinder scalability. For instance, VAE-based adaptive approaches exhibit cubic-time complexity *O*(m^3^) in Kalman filter-based update mechanisms, which can significantly restrict throughput during periods of high network traffic [114]. Conversely, lightweight models for IoT and edge deployment achieve compact sizes of 276 KB or less but often compromise adaptability and comprehensive attack coverage for efficiency [129]. These observations highlight the inherent trade-off between resource consumption and performance in Online AutoML design.

### 8.3. Adaptation and Learning Challenges

Despite the objective of achieving full automation, many Online AutoML frameworks still depend on human intervention. Certain approaches require manual labelling of up to 1% of incoming data streams, which becomes impractical in high-volume or real-time network environments [60]. These dependencies undermine the potential for fully automated learning systems.

Transfer learning capabilities remain limited. Few frameworks explicitly support knowledge transfer across heterogeneous network environments or attack domains [118], thereby limiting adaptability when models are redeployed or encounter new operational contexts.

Although most frameworks claim to address concept drift, evaluations are typically conducted using static benchmark datasets rather than dynamic streaming data. This practice raises concerns about whether reported drift-handling capabilities reflect genuine robustness in real-world environments [61].

### 8.4. Evaluation and Validation Limitations

There is a persistent reliance on standard benchmark datasets such as CICIDS2017, UNSW-NB15, and NSL-KDD. Although these datasets enable comparison across studies, they fail to capture real-world traffic variability, evolving attack behaviours, or long-term concept drift. Notably, only two of the reviewed frameworks report evaluations under actual streaming conditions [62].

Additionally, many studies simulate online learning by incrementally processing static datasets instead of deploying models in uncontrolled, real-time environments with unpredictable traffic patterns [61]. This approach limits the practical validity of reported performance metrics.

Domain-specific frameworks present further constraints. For instance, Auto-CIDS is designed for vehicular CAN bus environments but is evaluated exclusively on simulated data, which limits the generalizability of its findings [118]. More realistic evaluation settings, using genuine streaming data, evolving attack patterns, and deployment-level constraints, are needed to validate whether the reported Online AutoML performance holds in practice.

### 8.5. Scalability and Deployment Challenges

Most existing Online AutoML frameworks are designed for specific network scales, ranging from device-level monitoring to enterprise environments that manage hundreds or thousands of sessions per second [114]. Few systems exhibit consistent adaptability across such diverse operational contexts.

Latency requirements add further complexity to deployment. Certain frameworks achieve sub-millisecond inference latency, as low as 0.8 ms, making them suitable for high-speed networks [129]. Others require up to 49 ms to support more comprehensive temporal modelling [116]. These trade-offs complicate the alignment of system capabilities with application-specific real-time constraints.

Furthermore, enterprise-grade solutions often require complex infrastructure, including Kubernetes orchestration, Kafka-based streaming pipelines, monitoring tools, and specialized hardware. These requirements result in considerable deployment and maintenance overhead [116].

### 8.6. Methodological and Technical Limitations

Hybrid learning paradigms that integrate supervised and unsupervised techniques have the potential to enhance zero-day detection. However, they introduce additional complexity in model coordination and hyperparameter tuning, as well as increased computational cost. These factors complicate AutoML search spaces and may destabilize online learning processes, culminating in difficulties with efficient real-time model deployment, where fast adaptation is essential. Explainability is largely overlooked, as most frameworks provide limited or no mechanisms for interpreting model decisions. This lack of transparency undermines analyst trust, especially when responding to novel or high-impact attacks. Additionally, significant variability in evaluation metrics, datasets, and experimental setups across studies hinders direct comparison and complicates the selection of suitable Online AutoML solutions for specific IDS deployment scenarios.

A primary research focus is the development of algorithms for online and streaming learning that are robust to adversarial interference or manipulation. Online learning techniques operate in dynamic contexts characterized by continuous data influx and concept drift, making them particularly vulnerable to threats such as evasion and data poisoning. Future research should focus on integrated frameworks that simultaneously address concept drift [7] and adversarial manipulation, enabling models to distinguish between natural distributional changes and malicious perturbations. Promising methodologies include the adoption of online anomaly detection [67], resilient loss functions, and adaptive model update techniques. Moreover, computational efficiency is a significant concern, as streaming systems require resource-constrained, low-latency approaches [7,67,205].

### 8.7. Integration of Differential Privacy or Certified Defences into AutoML Systems

Despite considerable progress in automating ML procedures, AutoML often lacks built-in mechanisms for privacy and robustness [206]. Integrating differential privacy [207] into AutoML pipelines is a complex yet crucial endeavour, particularly in sensitive and critical sectors such as finance and medicine. Future investigations should examine privacy-conscious model search methodologies that enhance performance under stringent privacy constraints. The incorporation of certified defences—techniques that provide explicit assurances against adversarial disruptions/perturbations—remains mostly unexamined in AutoML. This entails creating pipelines that autonomously select models based on both prediction accuracy and demonstrable resilience. Additionally, understanding the trade-offs among privacy, resilience, and performance in dynamic contexts is an important area of research [89,206,207].

### 8.8. Development of Benchmarks Combining Drift and Adversarial Attack Scenarios

Contemporary assessment frameworks often address either adversarial attacks or concept drift [7,208] in isolation, failing to capture real-world scenarios. A critical need exists for extensive benchmark datasets and assessment algorithms that concurrently integrate dynamic data distributions alongside adversarial perturbations. Subsequent research should focus on creating datasets that include controlled drift patterns—specifically, gradual, sudden, and recurring drift [7,208]—and incorporate adversarial attack scenarios (evasion and data poisoning). Furthermore, assessment metrics must include not only accuracy but also robustness, flexibility, detection delay, and recovery performance. Standardized benchmarks would enable uniform model comparison and promote the development of robust learning frameworks [7,173,208].

### 8.9. Comprehensive Public Library for Online AutoML

The domain would benefit significantly from a cohesive, publicly available library designed for Online AutoML. Most current AutoML systems [5,89] are tailored for static, batch-learning environments and do not accommodate streaming input, real-time adaptation, or adversarial robustness. A complete library must include modular components for data-streaming intake, incremental model training, drift monitoring, adversarial mitigation, and explainability. It must additionally incorporate benchmark datasets, standardized APIs, and assessment procedures to facilitate repeatability. Integrating visualization and interpretability capabilities would significantly improve accessibility and usefulness for a wider audience. This open-source platform would expedite innovation, facilitate cooperation, and connect theoretical inquiry with practical implementation [5,88,89]. Such a library would also help standardize experimentation across Online AutoML studies, reducing fragmentation and accelerating reproducible progress in IoT and EV charging security research.

## 9. Conclusions

This survey critically reviewed Online AutoML and adversarial machine learning in IoT and EV charging networks, highlighting both opportunities and persistent limitations. Specifically, the study established several potential attacks on the IoT and EV charging networks, and they include OCPP exploits, grid manipulation, billing fraud, device hijacking, botnets, weak authentication, MITM, replay, DoS, data leakage, data injection/false data attacks, insider misuse/credential compromise, and traffic pattern anomalies. While AI-driven security approaches have shown promise, existing solutions struggle with dynamic, high-velocity data streams, leaving Online AutoML pipelines effective solutions for IoT and EV charging networks. Despite the advantages of Online AutoML, it is vulnerable to evasion, poisoning, privacy, and system-level attacks. Traditional ML-based defences are often insufficient, highlighting a clear gap for adaptive, real-time security mechanisms.

This study discussed adversarial strategies such as adversarial training, detection/input transformation, discrete adversarial optimization, gradient obfuscation/masking, input sanitization and data provenance, moving target, federated, hardware-assisted, and ensemble and model randomization defences. Besides these defense strategies, the study highlighted that the relationship between attack types and deployment scenarios in IoT and EVCS reveals structural deficiencies. Feedback from Online AutoML can serve as a contamination conduit, with attacks manipulating this feedback stream and thus impacting both networks. Additionally, the FL architecture facilitates attack propagation, allowing for backdoor attacks and injection strategies. Moreover, logical assaults have tangible consequences, as failures in IoT or EVCS can damage equipment or destabilize power grids, underscoring the critical threat to physical infrastructure.

Current Online AutoML frameworks frequently lack system completeness, with fragmented integration of data preprocessing, feature engineering, algorithm selection, hyperparameter optimization, and continuous model monitoring. Most studies treat performance, security, and deployment constraints in isolation. Online AutoML research focuses on model adaptation and optimization, whereas adversarial machine learning studies emphasize attack detection without accounting for the dynamic, resource-constrained, and heterogeneous nature of IoT-enabled EV infrastructure. Simplified experimental settings further limit the applicability of existing approaches to high-velocity streaming data with strict latency requirements.

These limitations underscore a pressing research gap, i.e., the absence of unified, secure, and resource-efficient Online AutoML architectures capable of continuous learning, adversarial resilience, and real-world deployment in complex IoT and EV environments. Addressing this gap motivates future work in this area and thus allows the integration of other critical research directions when developing improved systems that address noted security issues.

## Figures and Tables

**Table 1:** Mapping EV charging threats to CWE, CVE, and MITRE ATT&CK techniques.

Threat	Relevant CWE	Example CVE(s)	MITRE ATT&CK Technique	ATT&CK ID
Unencrypted OCPP Sessions	CWE-319: Cleartext Transmission of Sensitive Information	CVE-2026-22539	Adversary-in-the-Middle	T1557
Denial-of-Charge via Communication Disruption	CWE-400: Uncontrolled Resource Consumption	CVE-2025-68133	Network/Endpoint Denial of Service	T1499
Exposed Debug Interfaces	CWE-1191: On-Chip Debug Interface With Improper Access Control	CVE-2025-26408	Public-Facing Device Exploitation	T1190/T1200
Unprotected Internal Buses	CWE-1199: Improper Restriction of Memory/Bus Operations	CVE-2022-1199	Local System Data Access	T1120/T1005
Insecure OTA Updates	CWE-494: Download of Code Without Integrity Check	CVE-2025-52263	Supply Chain/Ingress Tool Transfer	T1105/T1195
Buffer-Overflow and Logic Flaws	CWE-120: Buffer Copy Without Size Check	CVE-2026-22593	Privilege Escalation	T1068
Misconfigured Cloud Storage	CWE-200: Exposure of Sensitive Information	CVE-2021-39144	Cloud Data Exfiltration	T1567.002
Weak IAM and Broken Access Control	CWE-284: Improper Access Control	CVE-2023-28131	Account Access Abuse	T1078/T1098

**Table 3:** Comparison of user experience and efficiency across Online and AutoML frameworks.

Category	Framework	User Experience	Efficiency
AutoML Frameworks	Auto-sklearn [89]	Compatible with Python 3.7–3.9 has a drop-in scikit-learn estimator, and automatic model and hyperparameter selection through Bayesian optimization.	Standard AutoML baseline; may exhaust the whole budget and exhibit poorer performance under extensive or restricted time limits.
	TPOT [160]	Genetic programming is used via a simple Python API to improve ML processes.	Effective, although for medium-sized to massive datasets, it may be computationally sluggish.
	H2O AutoML [161]	Multi-language support for Python, R, with a web user interface (UI), i.e., H2O Flow; scalable and appropriate for business needs.	Besides scalability, it requires more resources than lightweight techniques, but it can run several models, including ensembles.
	AutoKeras [148]	Python AutoML for automation of DL or NAS.	Deep neural models may require substantial time and computational resources.
	Google Cloud AutoML [162]	Integration with the Google ecosystem allows drag-and-drop with its cloud-based UI.	Contains abstracted infrastructure, managed service, characteristic of cloud AutoML solutions.
	Auto-WEKA [163]	Automated hyperparameter and algorithm selection were developed using WEKA.	First notable AutoML framework; appropriate for hyperparameter/algorithm search.
	AutoGluon [145]	Robust, enables DL and stacking across different modalities, and is simple to implement with a single-line training script.	Fast and precise in a variety of benchmark operations; superior to several AutoML rivals.
Online Learning Frameworks	River [151]	Python module for streaming/online machine learning with adaptive/incremental learning; updates the model with individual samples sequentially.	Optimized for streaming and low-resource environments; adept at managing concept drift; rapid execution for single-sample updates.
	Massive Online Analysis (MOA) [164]	Java-based framework for stream clustering, classification, and evaluation.	Built for scalability in real-time, with extensive ability for mining of data streams.
	Scikit-Multiflow [165]	Python-based stream learning framework with incremental techniques, generators and drift detection.	Optimized for streaming data; facilitates real-time incremental adjustments.
	Stream-learn [154]	Python library for data stream analysis and drift assessment, accommodating various drift kinds.	Facilitates stream classifiers and generators, with an emphasis on evaluating drift and increments.
	Kafka-ML [159]	Supports online learning and continual model enhancement using streaming data (such as IoT pipelines).	Facilitates scalable machine learning implementations across streaming platforms.
	CapyMOA [153]	Python 3.10–3.12 API integrating MOA with PyTorch 2.12.0 and scikit-learn; includes workflows and tutorials.	Merges the speed of MOA with the flexibility of Python; supports workflows involving hybrid streaming designs.

**Table 5:** Comparison of adversarial ML studies by Online AutoML completeness.

Reference	Online Learning	Automated Adaptation	Drift Detection/Adaptation	Notes
Chen et al. [180]	Partial	Yes	Yes	Federated meta-learning with Reptile; performance-triggered adaptation
Mao et al. [177]	Yes	Yes	Yes	Online fine-tuning; adapts to evolving malware and detectors
Moskalenko et al. [203]	Yes	Yes	Yes	Explicit drift detection; self-distillation and hierarchical labelling
Amich and Eshete [197]	Yes	Yes	Partial	Moving target defence; time-based model pool updates
Feng et al. [186]	Yes	Yes	Partial	Stateful defence with adaptive hyperparameters (OARS)
Azizi Ariffin et al. [193]	Yes	Partial	Yes	Adaptive aggregation for federated IDS security
Ivgi and Berant [192]	Yes	No	Partial	Online adversarial augmentation without AutoML logic
Zhang and Huang [183]	Yes	No	Partial	Lifelong learning without automated optimization
Panigrahi et al. [181]	Yes	No	Partial	Sequential federated rounds; backdoor persistence
Gong et al. [59]	Yes	No	Partial	Real-time attacks under strict latency constraints
Liu et al. [176]	Yes	No	Partial	RL-based black-box evasion in network systems
Wang and Zuccon [184]	Yes	No	Partial	Poisoning in federated online learning-to-rank
Joe et al. [174]	Yes	No	Partial	Temporal evasion via future-state hallucination
Chiejina et al. [167]	No	No	Partial	System-level O-RAN analysis; interference-triggered response
Queyrut et al. [196]	No	No	No	TEE-based isolation without online adaptation
Apruzzese et al. [175]	No	No	Partial	Drift–adversary interaction analysis without mitigation

**Table 6:** Cybersecurity, Online AutoML architecture and AML issues.

Category	Issues	Brief Description
Cybersecurity Challenges	EV Security Challenges	Electric vehicle charging networks are susceptible to cyberattacks, including OCPP exploits/unauthorized access, grid manipulation, and billing malpractice, owing to their dependence on communication protocols (OCPP) and decentralized architecture.
	Vulnerabilities in IoT-Enabled EV Charging Systems	The incorporation of IoT devices introduces vulnerabilities, including inadequate authentication, unprotected communication, and limited device security, thereby increasing susceptibility to botnet and data-breach threats.
	AI-Driven Security Measures	These AI-powered security solutions facilitate smart threat identification and response; however, they also introduce novel threats, including adversarial attacks and model manipulation.
	Data and Concept Drift	These concepts imply that ongoing data streams in IoT and EV charging networks exhibit dynamic patterns, leading to changes in data distributions that can diminish model accuracy if inadequately managed.
Online AutoML Architecture	Automated Online Data Pre-Processing	This is described as real-time data cleansing, transformation, and normalization, which are essential for managing noisy, high-velocity streaming data before model training.
	Drift Detection and Adaptation Mechanisms	These mechanisms are required to detect changes in data distributions and to initiate model retraining/updates to maintain performance.
	Automated Online Feature Selection	This is the dynamic selection of significant features crucial for enhancing accuracy and effectiveness in ever-changing data contexts such as IoT or EV charging networks.
	Online Model Selection and Training	This is an adaptive selection and gradual model that enables continual learning from data streams without retraining from scratch.
	Hyperparameter Optimization in Streaming Context	This ongoing adjustment of model hyperparameters is essential to maintain optimal performance amid fluctuating data conditions and technological limitations.
AML Attacks & Defences	AML Attacks	These are various threats to contemporary ML models, including evasion, poisoning, and privacy-based attacks.
	AML Defences	These are a variety of defensive strategies proposed for safeguarding against AML attacks. Examples include MTDs, AT, hardware-assisted defences, DAO, FD, ensemble and randomization defences, GOM, etc.

## Data Availability

No new data were created or analyzed in this study. Data sharing is not applicable to this article.

## Abbreviations

The following abbreviations are used in this manuscript:

ADWIN	Adaptive Windowing
AML	Adversarial Machine Learning
APIs	Application Programming Interfaces
AT	Adversarial Training
AutoDP	Automated Data Preprocessing
AutoFE	Automated Feature Engineering
AutoML	Automated Machine Learning
CASH	Combined Algorithm Selection and Hyperparameter Optimization
CRC	Cluster-Repelling Contrastive
CSRF	Cross-Site Request Forgery
CW	Carlini–Wagner Attack
DAO	Discrete Adversarial Optimization
DDM	Drift Detection Method
DDoS	Distributed Denial-of-Service
DIT	Detection/Input Transformation
DL	Deep Learning
DNNs	Deep Neural Networks
DoS	Denial-of-Service
EDDM	Early Drift Detection Method
EMR	Ensemble and Model randomization
EV	Electric Vehicle
EVCS	Electric Vehicle Charging Station
EVCMSs	EV Charging Management Systems
EVCNs	Electric Vehicle Charging Networks
FD	Federated Defenses
FGSM	Fast Gradient Sign Method
FL	Federated Learning
GGA	Gender-based Genetic Algorithm
GOM	Gradient Obfuscation and Masking
HAT	Hoeffding Adaptive Trees
HBD	Hardware-based defenses
HPO	Hyperparameter Optimization
ICS	Industrial Control System
IDSs	Intrusion Detection Systems
IIoT	Industrial Internet of Things
Irace	Iterated Race
ISDP	Input Sanitization and Data Provenance
IT	Information Technology
MITM	Man-in-the-Middle
ML	Machine Learning
MOA	Massive Online Analysis
MTDs	Moving Target Defenses
NAS	Neural Architecture Search
NCEn	Negative Correlation Ensemble
OASW	Lightweight Adaptive System
OCPP	Open Charge Point Protocol
OT	Operational Technology
OOD	Out-of-Order
OTA	Over-The-Air
PGD	Projected Gradient Descent
PLC	Power Line Communication
PSI	Population Stability Index
RL	Reinforcement Learning
RFE	Recursive Feature Elimination
SMAC	Sequential Model-based Algorithm Configuration
SQLi	SQL Injection
TEEs	Trusted Execution Environments
TPOT	Tree-based Pipeline Optimization Tool
UI	User Interface
V2G	Vehicle-to-Grid
VAE	Variational Autoencoder
XSS	Cross-Site Scripting
